# Cognition-Emotion Dysinteraction in Schizophrenia

**DOI:** 10.3389/fpsyg.2012.00392

**Published:** 2012-10-12

**Authors:** Alan Anticevic, Philip R. Corlett

**Affiliations:** ^1^Clinical Neuroscience Research Unit, Ribicoff Research Facilities, Connecticut Mental Health Center, Department of Psychiatry, Yale University School of MedicineNew Haven, CT, USA

**Keywords:** schizophrenia, emotion, cognition, working memory, delusions, fronto-striatal circuits, amygdala, cortical disinhibition

## Abstract

Evolving theories of schizophrenia emphasize a “disconnection” in distributed fronto-striatal-limbic neural systems, which may give rise to breakdowns in cognition and emotional function. We discuss these diverse domains of function from the perspective of disrupted neural circuits involved in “cold” cognitive vs. “hot” affective operations and the interplay between these processes. We focus on three research areas that highlight cognition-emotion *dysinteractions* in schizophrenia: First, we discuss the role of cognitive deficits in the “maintenance” of emotional information. We review recent evidence suggesting that motivational abnormalities in schizophrenia may in part arise due to a disrupted ability to “maintain” affective information over time. Here, dysfunction in a prototypical “cold” cognitive operation may result in “affective” deficits in schizophrenia. Second, we discuss abnormalities in the detection and ascription of salience, manifest as excessive processing of non-emotional stimuli and inappropriate distractibility. We review emerging evidence suggesting deficits in some, but not other, specific emotional processes in schizophrenia – namely an intact ability to perceive emotion “in-the-moment” but poor prospective valuation of stimuli and heightened reactivity to stimuli that ought to be filtered. Third, we discuss abnormalities in learning mechanisms that may give rise to delusions, the fixed, false, and often emotionally charged beliefs that accompany psychosis. We highlight the role of affect in aberrant belief formation, mostly ignored by current theoretical models. Together, we attempt to provide a consilient overview for how breakdowns in neural systems underlying affect and cognition in psychosis interact across symptom domains. We conclude with a brief treatment of the neurobiology of schizophrenia and the need to close our explanatory gap between cellular-level hypotheses and complex behavioral symptoms observed in this illness.

## Introduction

Schizophrenia is perhaps one of the most complex neuropsychiatric illnesses (Walker et al., 2004) with a remarkably heterogeneous presentation (Peralta and Cuesta, 2001; Dutta et al., 2007). After more than 100 years of continuous research effort (Insel, 2010), it largely remains a puzzle and presents a major challenge for clinical neuroscience (Ross et al., 2006; Heckers and Konradi, 2010). Patients suffering from schizophrenia face a lifetime of disability across virtually all known higher cognitive functions (Reichenberg and Harvey, 2007) and specific affective processes (Aleman and Kahn, 2005; Kring and Moran, 2008) vital to social interaction (Ochsner, 2008), vocational function, and overall life outcome (Kee et al., 2003; Green, 2006). The earliest conceptualizations of schizophrenia emphasized fragmented thinking exhibited by patients and a “disconnect” between affective and cognitive functions (Bleuler, 1911). Modern diagnostic classifications typically conceptualize schizophrenia as a complex dimensional syndrome (Barch and Keefe, 2010) thought to arise from disturbances in distinct, but interacting neurotransmitter systems such as gamma-aminobutyric acid (GABA), glutamate, and dopamine (Krystal et al., 2003; Goldman-Rakic et al., 2004; Lewis and Hashimoto, 2007; Lewis et al., 2012), which compromise the function of distributed brain networks (Stephan et al., 2006). Dysregulation in such distributed neural systems is thought to give rise to diverse symptoms such as disturbances in perception (hallucinations), belief (delusions), emotional dysfunction (amotivation and anhedonia), as well as severe deficits in complex cognitive operations such as working memory, long-term memory, and executive functioning (Barch and Ceaser, 2012). Perhaps what is so puzzling about schizophrenia is that it affects broad and seemingly independent functions, producing neurocognitive disturbances that are far outside the realm of normal human experience (e.g., severe delusions). However, conceptualizing these emergent behavioral phenomena as “separate” often ignores how they may actually interact – a dichotomy particularly obvious in research on affect and cognition (Pessoa, 2008). Although we base our clinical diagnosis on the overt behavioral deficits in these seemingly “independent” processes, we have a growing understanding that the human brain is not simply carved into modules that give rise to “emotional” vs. “cognitive” deficits in mental illness (Pessoa, 2008), as might be argued by lesion accounts. Thus, examining how these vital and complementary processes interact is critical for our complete characterization of the emerging symptom profile in schizophrenia.

Only in recent decades has our understanding of basic brain function evolved to elucidate the critical interplay of brain computations involved in “hot” affective processes (e.g., fear) and traditionally “cold” cognition (e.g., reasoning; Dolcos et al., 2004). That is, if we step beyond the study of mental illness, there is a long tradition in cognitive science largely devoid of any consideration for the role of affect (e.g., decades of research in memory, Tulving, 1972). Similarly, affective science has evolved in parallel, without a full integration into the emerging framework of cognitive science, until recently through the development of fields such as social, cognitive, and affective neuroscience. However, our academic divisions in research and the clinic do not always accurately cleave nature at its joints. Indeed, it has become widely accepted that brain regions involved in performing computations in the service of emotional and cognitive functions may be in a constant state of interaction depending on ongoing environmental and organismal demands (Pessoa, 2008). For instance, we now know that computations in the brain that give rise to fear responses are critically interwoven in the formation of memories (LeDoux, 2000).

Before we continue, we will briefly articulate some basic concepts and terminology that we will employ throughout the review. We will discuss findings related to the interaction of two traditionally independent, broad domains of function: emotion and cognition. Therefore it is important to briefly examine what we mean by “cognition” and “emotion.” Cognition typically refers to an ensemble of complex higher-level functions that are implemented across distributed brain networks involving mainly cortical regions; these include memory (Tulving, 1972), attention (Posner and Petersen, 1990), language (Petersen et al., 1990), and cognitive control (Miller and Cohen, 2001; Miller and D’Esposito, 2005). These higher-order functions are typically considered to be largely under volitional conscious control and are invoked in the service of some action or goal (Miller and Cohen, 2001; Miller and D’Esposito, 2005; Braver, 2012). On the other hand, it has been difficult to reach a universal consensus regarding the true definition of “emotion” (Lang, 2010) as this domain of function encompasses more elusive and diverse processes (reviewed in more detail below) taking place at vastly different temporal scales (Phillips, 2003; Phillips et al., 2003; e.g., from millisecond perception of fear-inducing stimuli to year-long mood states). Despite these challenges, some authors have attempted to define emotion as a set of computations originally implemented to motivate and mobilize action in the service of optimal survival (Lang and Davis, 2006; Lang, 2010; Lang and Bradley, 2010). From this conceptualization, emotion has been traditionally viewed as a broad set of evolutionary older functions, largely supported by subcortical and limbic structures (LeDoux, 2000), that perform computations mainly in the service of approach (in response to appetitive stimuli) or avoidance (in response to aversive stimuli) dispositions and actions, with more complex elaborations on these basic functions in higher-level organisms. This is not to say that emotion does not involve volitional and more complex processes in humans (e.g., guilt, jealousy, empathy Harvey et al., 2012). Indeed there are remarkable and complex elaborations on these evolutionarily older neural functions in humans (for a more comprehensive theoretical treatment of the functional utility and complexity of human emotion we refer the reader to prior reviews on this topic; Salzman and Fusi, 2010; Niedenthal and Brauer, 2012).

Nonetheless, here we conceptualize both emotion and cognition to represent broad functional domains with specific “sub-processes” implemented by select brain regions and/or networks of brain regions. Thus, it is important to note that it is less likely that there really is an interaction between “domains” of function (as a brain region does not “know” whether a computation being performed is specifically emotional or cognitive in nature), but rather between “sub-processes” (e.g., memory consolidation and detection of fear stimuli) that may be classified into one of these broader domains based on the functions they subserve. Furthermore, even specific “components” of cognition (such as working memory) are likely to encompass a complex interaction of lower-level processes that act in concert to orchestrate a set of computations in support of that aspect of cognition. For instance, computations within regions maintaining fidelity of memory traces, interacting with regions involved in suppressing external interference, could be considered as processes implemented by specific brain areas; however, they could both be considered as components of the general rubric of working memory (Jonides et al., 2008). Henceforth, when discussing processes, we will mainly be referring to a more specific set of computations implemented at the level of brain systems and/or network of regions. In contrast, when referring to domains of function (e.g., emotion and/or cognition or rather broad rubrics of cognition/emotion such as memory or mood) we will be referring to a higher level of analysis, which emerges as a result of the brain implementing and orchestrating specific processes.

Despite the different theoretical and empirical traditions giving rise to “affective” and “cognitive” neuroscience, we cannot continue to de-emphasize how emotion and cognition computations are intricately intertwined (Pessoa, 2008; Pessoa and Adolphs, 2010). For instance, affective information influences neural computations as early as basic visual processing (Bradley et al., 2003; Padmala and Pessoa, 2008). We contend that these interactions are particularly relevant for mental illness, which does not obey our, perhaps arbitrary, way of carving up emotion and cognition. In fact, we argue that serious mental illness such as psychosis involves deficits across both affective and cognitive operations, highlighting the need to understand their interactions not only in healthy human function, but how their breakdowns impact upon one another to affect behavioral deficits observed in mental illness. Nevertheless, despite evidence for severe emotional abnormalities in schizophrenia, research to date has investigated emotion in this illness largely in isolation from cognitive abnormalities (Aleman and Kahn, 2005), and emotional-cognitive interactions have yet to be explored systematically in patients suffering from schizophrenia (Kring, 2011). Of note, throughout the review we will use the term dysinteraction to emphasize that the relationships between cognition and emotion and their disruption in schizophrenia are not simply a unidirectional loss of function in a particular domain, but likely represent a dynamic interplay between functions.

In the present review we discuss three emerging research domains relevant to our understanding of schizophrenia that highlight this critical interplay of affective and cognitive operations and their breakdown. We will approach these topics from an affective and cognitive neuroscience perspective – that is we will highlight findings at the behavioral level and how they may relate to neural system-level findings that can be assayed with neuroimaging. First, we discuss the role of cognitive deficits and disruptions in neural systems responsible for context maintenance as in part contributing to motivational deficits in schizophrenia. Here we highlight findings suggesting that motivational abnormalities in schizophrenia may arise from the disrupted ability to “maintain” affective context over time to guide behavior. Through this perspective, we offer an example where a prototypical “cold” cognitive operation, namely working memory, may result in “affective” deficits in schizophrenia. Second, we review abnormalities in detection of salience that can manifest as heightened processing of non-emotional stimuli and inappropriate distractibility. Here we discuss emerging affective neuroscience findings in psychosis, which posit deficits in specific emotional processes, but not others – namely intact ability to perceive emotion “in-the-moment” but heightened reactivity to stimuli that ought to be filtered or ignored. Third, we highlight how breakdowns in learning mechanisms give rise to delusions, the fixed, false, and often emotionally charged beliefs that accompany psychosis. We offer an account for the role of affect in aberrant belief formation, mostly ignored by current theoretical models of delusions. Furthermore, we discuss how our continued understanding of schizophrenia and other mental illnesses depends on bridging our basic theoretical advances across affective and cognitive neuroscience to understand and ultimately treat complex neuropsychiatric disease. In turn, we argue that our evolving understanding of mental illness from this multidisciplinary perspective has the potential to cross-fertilize our basic understanding of human brain function. Finally, we will also attempt to link system and symptom-level findings to emerging cellular-level theories of neuropathology in schizophrenia (Krystal et al., 2003; Lewis et al., 2012; Marin, 2012). We argue that linking our cellular-level hypotheses with systems neuroscience findings and ultimately behavior is critical to close the explanatory gap that currently exists between our cognitive neuroscience evidence and hypotheses detailing synaptic pathology in schizophrenia research.

## The Role of Working Memory in Emotional Context Maintenance

Emotional deficits in schizophrenia are prominent. Since the seminal work of Bleuler (1911) and Kraeplin (1950), affective abnormalities have been considered a central component of schizophrenia symptomatology. However, the precise profile of emotional deficits in schizophrenia is complex. In fact, fully describing the range of affective abnormalities in this illness deserves a comprehensive treatment in itself and is beyond the scope of the present review (see Trémeau, 2006; Kring and Moran, 2008 for detailed discussion). Briefly, there is emerging evidence that patients with schizophrenia exhibit deficits in their expression of emotion (Krause et al., 1989; Berenbaum and Oltmanns, 1992; Kring et al., 1993, 1994; Mattes et al., 1995; Sison et al., 1996; Iwase et al., 1999; Cedro et al., 2001; Trémeau et al., 2005), recognition of emotional facial expressions and emotional classification (Mandal et al., 1998; Habel et al., 2000; Edwards et al., 2002; Kohler et al., 2003; Scholten et al., 2005), as well as anticipating hedonic experience (Gard et al., 2007). While all of these deficits are incapacitating and deserve research attention, here we focus on the third specific area of emotional dysfunction – namely the ability to guide behavior based on anticipated future rewards – a deficit which may, in part, underlie the negative syndrome and anhedonia (Barch and Dowd, 2010). A prominent feature of the negative syndrome in schizophrenia is the lack of motivation and inability to initiate appetitive goal-driven behaviors. Here we argue that this cardinal “emotional” symptom of psychosis can, at least in part, be conceptualized as a breakdown in the interaction of emotion and cognition – specifically as a deficit in representation of emotional context over time.

However, before we continue it is important to highlight one paradoxical finding that needs to be explained in the current framework: patients with schizophrenia seem to exhibit intact “in-the-moment” responses to emotional stimuli (Herbener et al., 2008). That is, when presented with emotionally laden stimuli, patients rate (Herbener et al., 2008; Kring and Moran, 2008), experience (Burbridge and Barch, 2002; Mathews and Barch, 2004), and activate neural structures (such as the amygdala) similarly to healthy controls (Anticevic et al., 2012b,c). This is further highlighted by a recent meta-analysis of the emotional neuroimaging literature in schizophrenia demonstrating little difference in amygdala signals in response to emotional probes relative to healthy controls (Anticevic et al., 2012c; but see a recent meta-analysis for a more extensive examination of other emotional tasks (Taylor et al., 2011)). Consistently, behavioral studies find that when presented with actual physical stimuli, patients tend to rate affective valence and arousal dimensions similarly as healthy controls (Herbener et al., 2008; Anticevic et al., 2012b). For instance, Herbener et al. (2008) asked that both patients diagnosed with schizophrenia and matched healthy controls provide “in-the-moment” ratings of arousal and valence dimensions of complex images that were pre-selected from the International Affective Picture System (IAPS). This study reported a very similar profile of arousal and valence ratings across both groups of subjects, an effect subsequently replicated in our own work (Anticevic et al., 2012b). These findings highlight that, when an affectively charged stimulus is readily available for sensory processing, the quality and the intensity of the emotional experience in patients seems comparable to that found in healthy populations (although more work is needed to fully rule out experimenter demand characteristics). As noted, this is somewhat paradoxical, because representations of those stimuli seem to break down over time and hence patients may not be able to use the affective information to guide behavior in a goal-directed fashion (Barch and Dowd, 2010).

Consistent with this hypothesis, Heerey and Gold (2007) demonstrated that patients diagnosed with schizophrenia exhibit deficits in their ability to translate subjective emotional experience into motivated behavior, particularly when relying on internal representations. These findings offer one source of support for the notion that motivated reward-driven behavior may be compromised in schizophrenia, even in the face of seemingly intact in-the-moment effects of positive and negative stimuli on behavior. This may occur due to a number of factors, one of which may involve a breakdown in context representations over time. Indeed, this is a thesis proposed by the authors (Heerey and Gold, 2007; p. 269): “Cognitive factors may also undercut the ability to couple motivational salience and behavior (Barch, 2005). For example, the degree to which an individual can activate a stimulus representation in working memory and use that representation to motivate behavior may prove important in understanding motivational deficits.” However, there is an important nuance – Heery and Gold did not observe a significant interaction between *Evoked* vs. *Representational* conditions across patients and control groups, suggesting that there may not be a substantial difference in these two processes. Nevertheless, there was a correspondence between *Representational/Evoked* behavior and emotional ratings, suggesting a larger discrepancy between the *Representational* condition behavior and ratings (as opposed to Evoked). The authors postulate this second effect may imply that (p. 273): “…patients have more difficulty generating behavior on the basis of internal representations than in the direct presence of an evocative stimulus.” The hypothesis that such affective deficits may be associated with problems in working memory was further bolstered by a significant relationship between both verbal and non-verbal working memory measures and the aforementioned effects. Together, these findings are in line with the proposal that guiding future reward-related actions may be compromised in part due to working memory deficits in schizophrenia.

Building on these insights, consider the following scenario: While patients may report that they enjoy a chocolate cake when they are consuming it, it seems that engaging in behaviors necessary to obtain the experience of the cake are compromised (Kring and Moran, 2008). Planning, purchasing, preparing, or baking the cake requires ongoing maintenance of contextual information regarding the food’s rewarding properties, which will ultimately guide the volitional pursuits over time that may lead to such a reward (in this case intake of appetitive food). While it is clear that an intact reward valuation system is necessary for this set of behaviors to take place (O’Doherty et al., 2002; Berridge, 2003, 2004; Barbas, 2007), this function in large part also depends on the intact ability to maintain an appetitive context over time – a process reliant on working memory (Barch et al., 2003) and cognitive control (Barch and Ceaser, 2012). This is where emotion and cognition may “dysinteract” in schizophrenia. This is not to say that deficits are completely absent in basic reward processing in schizophrenia, as evidence suggests this may be the case (Dowd and Barch, 2010; Nielsen et al., 2012). However, such a deficit in basic reward processing may be exacerbated via the inability to actively maintain rewarding information in working memory. In that sense, there may exist a “tension” in the transition from initial learning (perhaps reliant on basic reward processing) and using what is learned to guide complex behavioral routines (perhaps more reliant on working memory) that ultimately may become more ritualized and habitual (and hence freed from working memory demands). Breakdowns in this process have yet to be systematically explored.

There is strong evidence suggesting that patients with schizophrenia exhibit both behavioral and neural deficits in their ability to represent context over time (as demonstrated by findings from continuous performance tasks; Braver et al., 1999) as well as in their working memory operation (Lee and Park, 2005; Van Snellenberg et al., 2006). Some researchers would argue that these cognitive deficits are at the “core” of the illness because they emerge prior to the full syndrome (Cornblatt et al., 1999; Niendam et al., 2003), remain present across the life-span (Heaton et al., 2001; Irani et al., 2011), and are even observed in first-degree relatives of patients (Delawalla et al., 2006). They may pre-date the emotional deficits or perhaps contribute to their emergence. One possibility is that inability to maintain and update information in working memory in the service of guiding goal-directed behavior may in part contribute to and/or interact with motivational problems observed in this illness. In line with this hypothesis, using an elegant design, a recent fMRI study by Ursu and colleagues found an effect consistent with this possibility (Ursu et al., 2011). While in the scanner, Ursu and colleagues exposed subjects to affective or neutral pictures for a brief period followed by a delay interval during which subjects “maintained” the affective state. Following this delay all subjects were instructed to provide ratings of their emotional experience of the previously presented stimulus. Interestingly, during the initial stimulus presentation phase (i.e., while the physical stimulus was presented on screen), patients and healthy comparison subjects showed little difference in neural activity, as revealed by a direct contrast. In fact, both groups activated a distributed network of regions previously associated with processing affective stimuli, including the visual cortex, insula, thalamus, midbrain structures, and other regions (Kober et al., 2008). However, when required to “maintain” the affective content over the delay, individuals with schizophrenia exhibited marked reductions in signal levels across regions previously linked to cognitive control (e.g., dorso-lateral PFC; Wager and Smith, 2003; Owen et al., 2005). The lack of maintenance signals correlated with negative symptom severity in their sample. It is important to note that Ursu and colleagues found an association with negative and not positive stimuli – this limits the generalizability of their findings somewhat. Nevertheless, this general pattern of reduced prefrontal signal in the context of maintaining affective information is in correspondence with a body of evidence showing that schizophrenia is associated with reduced DLPFC signals during cognitive control and working memory tasks (Van Snellenberg et al., 2006). Indeed, in a recent investigation we demonstrated clear loss of prefrontal signal during the delay phase of working memory in a task context devoid of affect (Anticevic et al., 2011a), replicating and extending a large body of evidence suggesting lateral prefrontal abnormalities during working memory in schizophrenia (Glahn et al., 2005). It may be possible that the same deficit in the maintenance of context is at play irrespective of the type of information maintained in working memory. In other words, a deficit in a primarily “cold” cognitive operation may give rise to problems in maintaining affective representations.

If breakdowns in context maintenance indeed produce some affective abnormalities in this illness, then understanding and treating cognitive deficits may in part “rescue” some of the abnormalities we would traditionally consider as purely affective (e.g., lack of motivation). Indeed, there is evidence that suggests that patients with more superior cognitive performance on a working memory task manifest less of a difference between in-the-moment and delayed reports of emotion (Burbridge and Barch, 2002), suggesting that the deficit could be explained, at least to some extent, by breakdowns in cognitive control. However, there are still unresolved questions that future research may need to address:

First, there may be unique deficits in representing and maintaining affective information, stemming from breakdown in initial representations of context and information (e.g., encoding deficits) vs. deficits in representing and shielding such representations over time. In other words, forming accurate internal representations of an appetitive context (e.g., encoding novel stimuli) vs. actually representing and retrieving this context (e.g., maintenance of activity over a delay period) may constitute unique sources of deficits in schizophrenia (Lee and Park, 2005). It remains unclear what aspect of this emotion-cognition “dysinteraction” may be more severe. Recent work in working memory devoid of affect suggests that these abnormalities may be present across both formation and maintenance of active representations (Anticevic et al., 2011a).

Second, to what extent do such deficits in cognitive control, which may in part drive abnormalities in maintenance of emotional context, interact with reward learning mechanism? Patients may exhibit additional disruption in more basic reward processing/learning deficits (Barch and Dowd, 2010; Dowd and Barch, 2010). Together, these abnormalities may combine to exacerbate negative symptoms. In that sense, at the stage of the illness where anhedonia is already severe, we may be observing a confluence of problems in learning the rewarding properties of stimuli and the ability to represent those stimuli in mind over time to drive purposeful goal-driven behavior. We return to this question in more detail in the final section, where we argue that at least some symptoms of psychosis are associated with deficits in learning and interaction of learning mechanisms with affect. Nevertheless, it will be critical to further characterize the interplay and possible breakdown of reward responsiveness, learning, and cognitive control in schizophrenia. One target at the neural system level may be the interplay of prefrontal cortex and the ventral striatum, whose responsiveness to positive stimuli has been linked to levels of anhedonia symptoms in prior research (Dowd and Barch, 2010).

Third, no study has directly and systematically compared both cognitive and affective maintenance deficits in the same sample of patients. Prospective studies may want to examine whether the same pattern of results emerges: that is, are patients with the most severe loss of signal during “cold” cognitive operations such as working memory maintenance also exhibiting the most degradation of signal during maintenance of affective/reward-related representations, and whether this pattern relates to individual differences in symptoms. Similarly, it will be important to determine whether the loss of prefrontal signals during “affective” maintenance is predictive of subsequent behavior. For instance, one could envisage a cognitive-emotional fMRI paradigm that requires a given precision of affective context representation to guide subsequent task performance such as loss or gain of a reward. In doing so, future work could further link the lack of “maintenance” signals following affective/reward cues to deficits in future goal-directed behavior and negative symptoms.

Fourth, future research should also address why such a breakdown may be more manifest for approach-related (i.e., positive) stimuli vs. avoidance-related (i.e., negative) stimuli (Barch and Dowd, 2010). Ursu and colleagues found that the degree of DLPFC signal loss specifically in the positive condition was predictive of negative symptom severity. If the source of the deficit was in cognitive control regions, which may not be specific to any one valence in particular, then the specificity of the breakdown in reward-related behavior (rather than defensive ones) needs to be explained and linked to possible striatal deficits. Perhaps, as Ursu and colleagues argue, converting active affective representations into motivated behavioral pursuits requires a different dynamic interplay between PFC and reward-related neural circuits (e.g., ventral striatum and orbitofrontal cortex). Future studies should further elucidate these possibilities using convergent task-based activation and functional connectivity methods that probe disruptions in distributed fronto-striatal circuits as a function of valence.

Fifth, prior work suggests that some of the observed differences in reward responsiveness and representation are related to individual differences in symptom severity (Dowd and Barch, 2010; Ursu et al., 2011; Nielsen et al., 2012). These findings highlight that observed deficits may not constitute a stable trait of the schizophrenia “diagnosis,” but instead suggest that the level of the state (i.e., negative symptoms) may be related to the severity of regional disturbance and the dysinteraction between the aforementioned distributed prefrontal-striatal systems. Given this clinical heterogeneity, we suggest that emotion-cognition dysinteraction in this case may arise due to inadequate striatal reward representation and/or abnormal context representation in areas such as DLPFC. That is, these may be dissociable processes, at least to a certain extent. One way to test this possibility is to examine both striatal and cortical function across reward and executive tasks in the same well-powered sample. Indeed, as suggested by Dowd and Barch (2010), it will be critical for future studies to employ adequately powered samples to take into account possible individual differences. The importance of such a “dimensional” approach is further highlighted by recent National Institute of Mental Health efforts to link severity of system-level disturbances to emergent behavioral symptoms and underlying genetic risks (Insel and Cuthbert, 2009; Insel, 2010).

Finally, it remains to be determined whether the same hypothesized underlying neurotransmitter and cellular-level neuropathology is indeed producing deficits observed in the maintenance of “affective” vs. “cold” representations over time. There is evidence for striatal dysfunction in schizophrenia contributing to reward representation abnormalities, which may in part be driven by dopaminergic disruption (Laruelle et al., 1995, 1999). In turn, cognitive control deficits in schizophrenia are also associated with dopamine imbalance (Abi-Dargham et al., 2002), but may also arise due to NMDA and/or GABA pathology (Krystal et al., 2003; Lewis et al., 2012). Future work will need to characterize the role of complex system-level neurotransmitter interactions in these co-occurring processes. One technique that may help us understand the source of these neurochemical deficits mechanistically involves pharmacological manipulations in healthy adults (Krystal et al., 1994). These manipulations impact upon different neurotransmitter systems thought to be implicated in cognitive/affective disturbances in this illness (Corlett et al., 2006), which can be elegantly combined with human functional neuroimaging (Honey and Bullmore, 2004). For instance, a recent study successfully combined two convergent pharmacological challenges thought to impact on dopamine vs. NMDA neurotransmitter systems through the combined use of amphetamine and ketamine administration (Krystal et al., 2005). Krystal and colleagues showed that ketamine induced cognitive and negative symptoms, whereas this effect was ameliorated by amphetamine. In contrast, both manipulations exacerbated positive psychotic symptoms, but the effects were not interactive. This study highlights how pharmacological approaches can begin to elucidate and dissociate the role of complex and interacting neurotransmitter systems in the formation of behavioral symptoms such as emotional (e.g., negative) and cognitive deficits. Furthermore, as noted, such pharmacological manipulations can be successfully combined with human functional neuroimaging to experimentally probe whether a common neurotransmitter pathway may be involved in these seemingly distinct manifest symptoms (Honey and Bullmore, 2004). Through this approach, we may be able to mechanistically examine neurotransmitter sources of observed deficits across affective and cognitive domains in a controlled experimental setting in healthy volunteers. The challenge facing the field is to elucidate how breakdowns in cognitive/emotional processes interact in schizophrenia, but also to move toward a final common neurobiological pathway that explains deficits across processes in such a way that treatment of both deficits can be applied, perhaps concurrently. Dowd and Barch (2010) have hypothesized that neuropathology in prefrontal-striatal signaling may in part underlie these deficits. We further discuss the need for such translation in the final section.

In summary, in the proceeding section we highlighted how a dysfunction in a “cold” cognitive process may in some ways give rise to deficits that compromise affective/reward processing. In the following section we focus on a similar possibility in the domain of immediate perception of salient and non-salient stimuli in the environment – particularly from the perspective of sensory filtering.

## Aberrant Salience in Psychosis – Over-Responsiveness to “Neutral” Stimuli

Another seemingly paradoxical finding in the study of emotion, cognition, and their interaction in schizophrenia relates to patients’ perception of “neutral” stimuli (i.e., stimuli that are not perceived as salient by healthy participants). A number of different lines of evidence suggest that patients with schizophrenia experience a state of “aberrant salience” marked by a blurred distinction between relevant and irrelevant stimuli in the environment (Gray et al., 1995; Gray, 1998; Kapur, 2003; Corlett et al., 2009a). Preclinically, this state has been captured as a weakening of the phenomenon of latent inhibition; normally, stimuli that have repeatedly been experienced as inconsequent receive less attention and do not enter as readily into associative relationships compared to non-pre-exposed stimuli. However, in the context of psychotomimetic drugs (O’Tuathaigh et al., 2003) and endogenous psychosis (Gray et al., 1991) the phenomenon of latent inhibition is weakened and irrelevant stimuli garner attention. This state of “aberrant salience” is a complex phenomenon, possibly mediated by breakdowns in glutamatergic (Corlett et al., 2010a) and dopaminergic signaling (Howes and Kapur, 2009) (see Box 1), and a number of models have postulated neurobiological mechanisms that may explain this effect (Kapur, 2003; Corlett et al., 2009a; Howes and Kapur, 2009). In this context, it is important to briefly distinguish between psychosis and schizophrenia: the former being a set of symptoms describing a state and the latter representing the syndrome or a theoretic construct used to label the constellation of manifest symptoms (Walker et al., 2004). While we argue that this deficit of aberrant salience is present in schizophrenia patients, it is proposed that it might be particularly exaggerated during acute psychosis (Gray, 1995, 1998; Gray et al., 1995; Kapur, 2003). An influential model originally proposed by Jeffrey Gray, David Hemsley and colleagues (Gray, 1995, 1998; Gray et al., 1995) and reiterated by Kapur more recently (Kapur, 2003; Kapur et al., 2005) suggests that a primary disruption in dopaminergic signaling in the mesolimbic pathway may result in exaggerated dopaminergic tone, which impacts upon prefrontal-striatal circuits. Indeed, Kapur states “…dopamine mediates the conversion of the neural representation of an external stimulus from a neutral and cold bit of information into an attractive or aversive entity.” By assigning salience, via elevated ventral striatal dopamine, patients may imbue “emotionality” and meaning to random and/or typically irrelevant events.

Box 1 **Dopamine, salience, and psychosis.**

As noted, multiple neurotransmitter systems have been implicated in schizophrenia, with dopamine playing a central role in aberrant salience. Therefore, it is critical to briefly consider dopamine in the present account and in particular medication targeting dopamine neurotransmission. The influential aberrant salience model was developed as a means of explaining to patients why, when they take their D_2_ dopamine receptor blocking medication, do their delusions resolve (Kapur, 2003). Here we briefly consider the intersection between D_2_ receptor blocking drugs, cognition, emotion, and symptoms. Certain negative symptoms like anhedonia and apathy may pre-date illness onset and are present in unaffected and un-medicated relatives (Chen et al., 2009), hence not all emotional deficits in schizophrenia are iatrogenic. However, attenuating dopamine transmission can curtail some motivated behaviors (Dickinson et al., 2000). While D_2_ receptor antagonists ameliorate positive symptoms, their site of action may contribute to some of the anhedonia and amotivation observed in schizophrenia, because blocking dopamine transmission (particularly in the limbic striatum/nucleus accumbens (Beninger, 1983) can decrease motivation (Dickinson et al., 2000), perhaps explaining some side effects of D_2_ blockers like loss of libido. Future work should continue to explore effects of D_2_ blockers on emotional/cognitive processes in schizophrenia.

In terms of the underlying functional neuroanatomy, the site at which D_2_-blocking antipsychotic drugs exert their effects was always assumed to be the nucleus accumbens or ventral striatum, a key region implicated in the ascription of aberrant salience (Kapur, 2003; because accumbens dopamine release was related to the ascription of motivational significance or salience to drug-related cues in preclinical models of addiction (Robinson and Berridge, 2001)). However, the Positron Emission Tomography (PET) data in patients with psychosis and individuals in the prodromal stages of psychosis point instead to the associative striatum or head of the caudate nucleus as a possible site of dopamine pathophysiology that may relate to psychosis, as well as a key target for D_2_ medication effects (Howes et al., 2009). Hence, D_2_ abnormalities in associative striatum (as opposed to ventral striatum) may be associated with positive symptoms whereas pathophysiology in the accumbens coupled with iatrogenic effects might underpin the negative symptoms (Kegeles et al., 2010). In support of the latter point, Juckel and colleagues employed a monetary incentive delay task in which a visual cue signals the requirement to emit a speeded instrumental response to gain (or avoid losing) money (Knutson et al., 2000). They found aberrant striatal signaling in patients with schizophrenia, indicative of a “blurring” of the distinction between salient and non-salient events in the striatum. This effect was significantly related to negative but not positive symptoms (Juckel et al., 2006a). A follow-up study suggested an iatrogenic origin for the effect (Juckel et al., 2006b); when patients were switched from typical D_2_-binding drugs to atypical antipsychotics with significantly less D_2_ receptor affinity, the striatal aberrations appeared to normalize (Schlagenhauf et al., 2008). Together, these findings highlight that carefully considering the site, source, and symptom relevance of D_2_ antagonist effects is crucial to discerning their role in this illness as well as contributions to cognitive/affective dysfunction.

Nevertheless, it is important to consider alternative mechanisms to D_2_ dysfunction in psychosis: D_2_ receptors and dopamine dysfunction may not be the only neural mechanism with relevance to psychotic symptom formation. In the context of a transient and reversible psychotomimetic ketamine infusion in healthy volunteers, inappropriate prediction error signals have been observed in cortex and these signals were associated with aberrant salience experiences and delusion-like ideation (Corlett et al., 2006). Indeed, dopamine does not appear to play a significant role in the acute effects of psychotomimetic compounds that impact the NMDA receptor. For instance, effects of ketamine (an NMDA antagonist) are not blocked by haloperidol (Krystal et al., 1999) and the PET data on raclopiride binding under ketamine are mixed (Kegeles et al., 2000). On the other hand, the psychotomimetic effects of ketamine are attenuated by lamotrigine, a drug that blocks presynaptic glutamate release (Anand et al., 2000). Hence, unregulated glutamate release may be another mechanism driving aberrant prediction error and psychosis. In contrast, dopamine may be more relevant for the longer lasting effects of repeated ketamine exposure and the emergence and maintenance of positive symptoms (Corlett et al., 2011). These hypotheses should be the focus of future empirical investigation to more fully characterize dopaminergic/glutamatergic mechanisms in psychotic symptoms.

A recent framework proposed by Corlett and colleagues argues for a disruption in prediction error mechanisms, mediated by NMDA and AMPA currents, that may operate during learning and belief formation – we discuss this framework extensively in the final section. The latter model argues that healthy perception and belief proceed via the generation of expectations, which are compared to incoming experiences. If expectations are not properly specified or sensory inputs misprocessed, prediction errors result. These prediction error signals guide learning and update future expectations or regulate the cancellation of sensory inputs (Corlett et al., 2009a,b, 2010a). Prediction errors impact learning directly, forging the formation and strengthening of associations (Rescorla and Wagner, 1972), and indirectly, through the reallocation of attention toward potentially explanatory stimuli (Pearce and Hall, 1980). Aberrant prediction error signals, registered independent of cue or context, drive the assignment of salience to random or otherwise ignored internal and external events, a state which may ultimately result in development of delusions. We will return to the commonalities and differences between these accounts as well as the empirical data on dopamine function in patients and the potential effects of antipsychotic medication on salience, emotions, and learning.

We highlight patterns of findings from apparently disparate lines of work that offer evidence consistent with this aberrant salience effect. We present the results from four separate experimental domains: studies of fear conditioning, learning, and reward processing, as well as the filtering of distracting information during working memory. Findings across these domains could be conceptualized broadly under this rubric of abnormal assignment of salience and/or deficits in suppressing salient stimuli. Moreover, these effects may result in an experience of neutral or irrelevant stimuli as meaningful and affectively laden, possibly manifest ultimately as an acute state of psychosis. If this indeed is the case, then during states of elevated psychosis, patients may by definition exhibit abnormalities in their emotional function driven by exaggerated responsiveness to events/stimuli that are typically not perceived as such by healthy individuals (Kapur, 2003).

**Figure 1:**
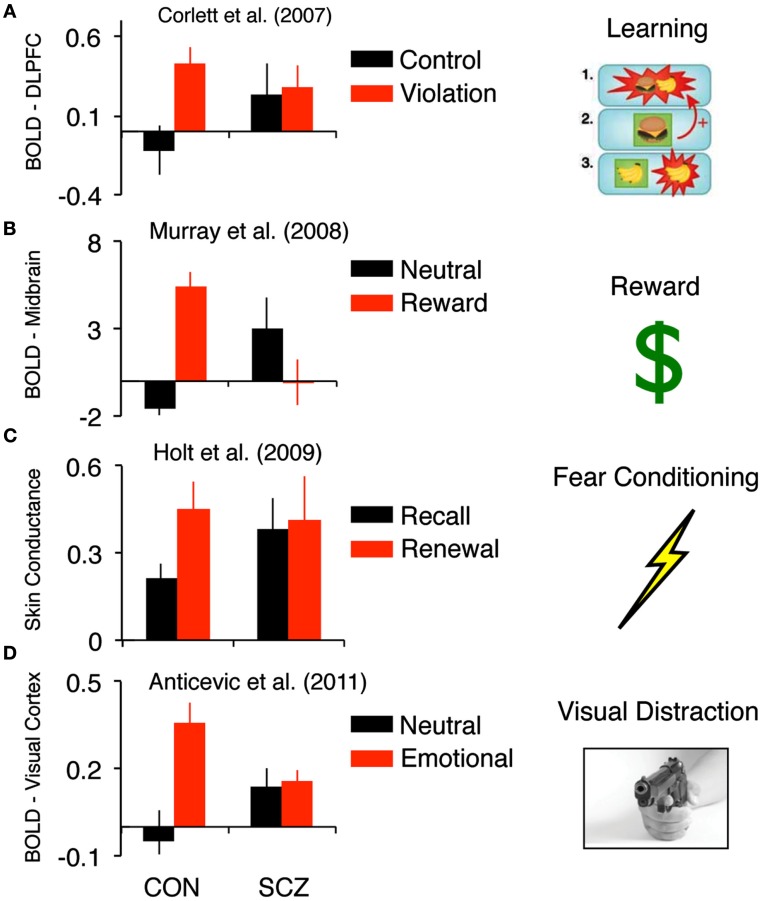
**Findings across different experimental contexts that highlight inappropriate responsiveness to “neutral” information in schizophrenia**. **(A)** Corlett et al. (2007a) showed, in the context of “cold” associative learning, that events that did not violate expectation were associated with increased DLPFC signals in schizophrenia patients relative to healthy controls. **(B)** Murray et al.’s (2008) findings highlight, in the context of reward learning, that non-rewarding events were associated with increased striatal signals in schizophrenia patients relative to healthy controls. **(C)** Holt et al.’s (2009) results show aberrant skin conductance fear responses to cues that should be neutral (extinguished), in line with the pattern of responses observed in the brain during causal learning and learning about monetary rewards. **(D)** In a delayed working memory study faced with distraction, Anticevic et al. (2011b) found that patients were distracted by non-salient distraction, which was also associated with increased signals in basic visual regions in patients, particularly when distracted. Together, these findings suggest, across different experimental contexts and measures, that there may be evidence for increased responsiveness to “neutral” stimuli for which healthy controls respond to as less salient.

The first study we highlight as reporting this effect was conducted by Corlett et al. (2007a). The authors studied the process of aberrant association formation in first-episode psychosis. They examined brain responses, using fMRI, during learning of associations and violation of those associations in patients compared with matched control subjects. Behaviorally, both groups of subjects acquired the associative relationships presented to them. This was critical as any demonstration of brain or behavioral differences during violation of expectation hinge upon both groups being able to learn the basic associations. In the context of associative learning it is possible to “violate” a learned expectation – this generates a prediction error, which can be conceptualized as a signal of salience or novelty (i.e., something in the environment does not match the organism’s prior expectancy). Corlett and colleagues compared brain responses to such surprising events to those that occurred in response to events that confirmed predictions. Healthy subjects showed a robust difference in right lateral PFC response between the two conditions (Corlett et al., 2007a). However, there were group differences between patients and controls in right lateral PFC response (see Figure 1A). The pattern observed in patients can be best described as a “blurring” in the distinctiveness of responses to events that violate the expected association and events that confirmed expectations. Crucially, those patients in whom this effect was most pronounced reported the most severe delusions.

Murray et al. (2008) illustrated a highly similar phenomenon in the context of reward learning (as opposed to the relatively “cold” association process highlighted above) in a case-control design with first-episode psychosis patients. Subjects were asked to select one of two visually presented stimuli and observe the outcome of their choice: either a financial reward or no reward (neutral feedback). A computational analysis estimated prediction error from subjects’ behavioral choices and aligned these estimates with subjects’ brain responses to rewarding and neutral events. Comparing these two conditions in control subjects revealed a canonical set of brain regions associated with reward processing including the midbrain and ventral striatum. Patients with psychosis showed prediction error brain responses in the midbrain and striatum to neutral events that should not be salient. Again, this pattern of experimental findings highlights a blurring of the distinction between responses to events that should be important and those that were relatively unimportant (Figure 1B). This pattern of results was also replicated during aversive learning about cues that predicted the delivery of mild transcutaneous shocks whereby inappropriate midbrain prediction error signals in a psychotic patient group correlated with delusion severity (Romaniuk et al., 2010). Inappropriate striatal prediction error signals to neutral cues that do not predict electric shock have also been observed (Jensen et al., 2008), however, these signals did not predict delusion severity.

The pattern of over-responsiveness to relatively neutral events compared to truly salient cues has been seen not only in brain signals but also in the peripheral physiological responses to those cues that predicted aversive electrical stimuli. Aversive events and the stimuli that predict them induce a sweating response that changes the skin conductance, which can be measured with electrodes. This skin conductance response is reflective of aversive learning or fear conditioning, as well as its subsequent correlation with previously neutral stimuli (arising through learning) when those neutral stimuli predict the aversive events. Patients with schizophrenia show a noisy skin conductance response consistent with an aberrant salience response (Gruzelier, 1976; Holt et al., 2009), which again correlates with delusion severity (Holt et al., 2009). Holt and colleagues used this response to explore extinction learning. In brief, extinction involves new learning such that a once salient stimulus, which was predictive of an important outcome (in this case transcutaneous electrical shock), is no longer predictive. Patients with schizophrenia can engage in extinction learning – their skin conductance responses to extinguished stimuli indeed decrease (Holt et al., 2009). However, that learning does not consolidate, such that when tested 24 h later, patients with schizophrenia showed fear responses to the cue that should now be neutral, in line with the pattern of responses observed in the brain during causal learning and learning about monetary rewards described above (Corlett et al., 2007a; Murray et al., 2008; Figure 1C).

In the final example we discuss a study by Anticevic et al. (2011b) examining the effects of interference during a delayed visual working memory task in patients with schizophrenia and matched healthy control subjects. While in the scanner, subjects performed a 2 h task that contained trials with no distraction or a distracter presented during the working memory delay phase. Of note, the distracter intensities were manipulated to examine the effects of different levels and types of interference on the maintenance of information in working memory in schizophrenia (i.e., they were either a complex neutral picture, complex emotionally negative picture, or a task-relevant distracter matched to the original memory set). The behavioral results demonstrated that patients were distracted irrespective of distracter type. Importantly, this effect was present even though the two groups were matched for performance when no distraction occurred (Anticevic et al., 2011b). Second, fMRI results showed that patients failed to recruit a region of the right dorso-lateral prefrontal cortex (DLPFC) specifically in response to distraction (again irrespective of distracter type). The degree of DLPFC recruitment correlated significantly with working memory accuracy for controls, specifically during the distracter condition – illustrating the functional relevance of this DLPFC response, in line with prior work implicating this region in filtering of distraction (Postle, 2005). No such relationship was observed for patients, supporting the hypothesis of a failure in distracter filtering. Together these findings show that even stimuli that are perceived as less salient and successfully “filtered” by healthy controls present a source of interference for patients. Furthermore, in complement to the observed prefrontal deficits, we identified a set of regions in primary visual and association cortices for which patients exhibited elevated responses, particularly when distracted by neutral information (Figure 1D). In other words, when both patients and controls were distracted (by examining incorrect trials specifically) we found an fMRI pattern in line with those described above – namely patients showing over-responsiveness in sensory regions when controls exhibited no such response.

These convergent findings suggest that patients respond abnormally to stimuli that are not perceived as salient by healthy controls. Aberrant salience experiences are inherently anxiogenic and hence demand explanation. Next, we focus on that explanatory process and how it might culminate in delusion formation.

## The Possible Role of Affect in Formation and Maintenance of Delusions

Delusions, the fixed, false beliefs present in schizophrenia, have been considered intractable to scientific inquiry (Jaspers, 1963). Recently, via cognitive neuroscience we have made strides in our empirical understanding of aberrant belief formation (McKay et al., 2007a; Fletcher and Frith, 2009; Coltheart, 2010; Corlett et al., 2010a; Coltheart et al., 2011). Theoretical models grounded in translational cognitive neuroscience suggest that delusions could result from disrupted brain mechanisms of predictive learning (Corlett et al., 2007b, 2010a; Fletcher and Frith, 2009). However, these accounts have mostly ignored the potential role of affective contribution to the process of belief formation and maintenance (Fotopoulou, 2010). Here we briefly expand existing theoretical accounts of delusion formation to incorporate the role of affective signals.

What role might affect play in delusion formation? As suggested, aberrant salience experiences are anxiogenic, since they are surprising and therefore demand explanation (Kapur, 2003). For instance, in uncertain conditions and stressful situations, people are likely to experience apophenia (Conrad, 1958) – perceiving structured meaning in meaningless noise (i.e., seeing and hearing things that are not actually there; Whitson and Galinsky, 2008). Furthermore, according to two-factor theories of delusions (Coltheart et al., 2011; see below), the Capgras delusion (believing that one’s relatives have been replaced by impostors) and Fregoli delusion (the belief that strangers on the street are one’s relatives in disguise) may be in part driven by a lack of predicted emotional response and an excessive emotional response, respectively. This possibility is supported by studies of the galvanic skin response (GSR) in Capgras sufferers who adopt the belief following head injury – they do not show normative skin conductance increase (sweating) when confronted by a close family member (Ellis and Young, 1990). This lack of familiarity response could be surprising and thus may demand explanation. In turn, such aberrant emotional responses could result in the impostor belief being formed (Corlett et al., 2010a,b). Based on this account, the Cotard delusion (the belief that one is dead) may be associated with an attenuated affective response to otherwise salient cues (Ramachandran and Blakeslee, 1998). Further supportive data for the role of affect have yet to be acquired for individuals suffering the Cotard or Fregoli delusions. Nevertheless, the noted finding in the context of the Capgras syndrome (Hagen, 2008) supports the hypothesis that emotional processes play a role in at least some delusions. In the following sections, we discuss how such processes could interplay with established models of aberrant belief formation.

We consider the learning model in the context of prior accounts that explain delusions in terms of aberrations of emotion, motivation, and desire (McKay et al., 2007a,b; Fotopoulou, 2010). Central to these accounts is the notion of self-deception (Trivers, 1985; Hagen, 2008; Mijovic-Prelec and Prelec, 2010), whereby patients with delusions hold their beliefs to maintain a model of the world that is not veridical but rather conforms to internally held views and thus avoids the negative emotions associated with discrepant perceptions (McKay et al., 2007a,b; Fotopoulou, 2010). These theories argue for a multi-agent conception of self (Mijovic-Prelec and Prelec, 2010), postulating an actor that supports self-deceiving beliefs, and a more objective critic that infers the appropriateness of those beliefs. We argue that such multi-controller models in the context of delusions are highly consistent with multi-controller models of instrumental learning from formal animal learning theory, which has been linked to delusion formation (Sutton and Barto, 1998; Daw et al., 2005). Later, we discuss how motivational processes may interact with the actor and critic.

Multi-controller models are accounts of instrumental learning (learning the consequences of one’s actions in the environment) from the psychology literature (Dickinson and Balleine, 1990) and the computational reinforcement learning literature (Daw et al., 2005). Such models posit more than one system (controller) that can guide instrumental action and choice. Each controller is hypothesized to use a different representation or metric to guide behavior. For example, psychologists have defined a goal-directed controller of instrumental action that is driven by the value of the outcome being learned about and mediated by stimulus-response-outcome associations. In contrast, they also postulate a more habitual controller that employs stimulus-response associations (and is hence insensitive to the value of the outcome being worked for) may also control instrumental behavior. With over-training, habitual control takes over such that, even when the outcome is devalued (e.g., by poisoning or satisfying the subject by free-feeding), animals continue to respond for the outcome (Adams and Dickinson, 1981). Functional neuroimaging data have demonstrated consistent effects in humans and linked habitual control with dorsal striatal signals (Tricomi et al., 2009). Some theorists posit that both controllers are present simultaneously and compete to guide behavior (Daw et al., 2005), which seems to be the case since manipulations can be made to switch habitual animals back toward goal-directed responding (Hitchcott et al., 2007). One possibility is that the competition is based on whose predictions are least uncertain (Daw et al., 2005). Therefore, prediction error (and the uncertainty with which it is associated) can bias toward a particular controller of behavior (Butts, 1998). If such signals are generated inappropriately and internally, they could contribute to development of aberrant beliefs, which are then maintained as cognitive habits. In schizophrenia, due to abnormal distractibility, cognitive impairment, and susceptibility to stress, corrective mechanisms that involve computationally intensive reasoning and evaluation may not be able to ameliorate fallible maladaptive beliefs (delusions; Mishara and Corlett, 2009). Furthermore, with excessive ruminative self-reinforcement (Eisenhardt and Menzel, 2007) the inflexible, self-deceptive habitual system may gain control despite corrective feedback (Corlett et al., 2009b). Critically, motivational factors may play a key role in this process.

Specifically, motivational processes could be considered in the domain of paranoid and persecutory beliefs (Kaney and Bentall, 1989, 1992; Kinderman and Bentall, 1996; Bentall et al., 2001) that defend against low self-esteem (McKay et al., 2007a,c). One possibility is that individuals with persecutory delusions have high overt self-esteem and relatively low unconscious, covert self-esteem (Kinderman, 1994; Kinderman and Bentall, 1996), which seems to be the case in patients with delusions (McKay et al., 2007c). Kinderman (1994) showed that individuals with persecutory delusions (a cardinal example of paranoia) exhibited attentional bias toward words related to low self-esteem while simultaneously reporting high self-esteem on an explicit self-rating task. Using the Emotional Stroop task (in which there is a response cost for naming the color of emotionally salient words), Kinderman found that individuals with persecutory delusions showed slowing to name the colors of negative self-descriptors. In contrast, on an overt measure of self-esteem, the same individuals exhibited higher self-esteem, explicitly rating themselves more highly on positive vs. negative adjectives. Kinderman concluded that this discrepancy between covert and overt self-esteem might reflect the impact of a defensive process that culminated in the formation of persecutory delusions. We acknowledge this is indirect evidence, but it offers some support for the role for self-esteem and motivational factors in persecutory delusion.

To further investigate the role of self-esteem in persecutory delusions, McKay et al. (2007c) used the implicit association test (IAT), demonstrating a link between implicit self-concepts, covert self-esteem, and persecutory delusions. The IAT measures automatic associations between concepts (Greenwald et al., 1998). Typically participants need to give two different responses to words from pairs of categories (e.g., press right hand button for words that fall into *flower* or *pleasant* categories and the left button for words that fall into the *insect* or *unpleasant* category). The association between the concepts that are paired together is hypothesized to be stronger the faster the subject responds. McKay and colleagues used *self*, *other*, *pleasant*, and *unpleasant* categories. As shown previously, the ease of making judgments when the *self* and *pleasant* categories are combined under one response rule can be used as a measure of implicit self-esteem (Greenwald and Farnham, 2000). Subjects also completed a number of overt self-relevance rating measures. Patients with current persecutory delusions showed a weaker implicit association between self and positive compared with depressed and remitted control groups, even when covarying for depressive symptoms. In contrast, when accounting for depression there were no differences between groups on overt self-esteem. The difference between implicit and overt self-esteem measures in paranoid patients is consistent with a possible defensive function – paranoid delusions may in part be related with the motivation to maintain self-esteem. One emphasis of both motivational/self-protective processes and learning accounts is the *need for closure* (Kruglanski et al., 1993; Kruglanski and Webster, 1996) – a motivational construct, associated with a preference for certainty and predictability (McKay et al., 2007a,c). Patients with persecutory delusions have higher need for closure (Bentall and Swarbrick, 2003). In the learning account, need for closure represents the drive to minimize prediction error and infer a model of the internal/external world that, although maladaptive and self-deceptive, reduces uncertainty (Mishara and Corlett, 2009). Across both accounts, such self-deception may be adaptive in that it allows the individual to engage in the world despite contradictory experiences (McKay and Dennett, 2009).

Another intersection of learning-based models of belief formation and emotion is fear. Most reinforcement learning models relevant to delusions center on reward processing, and the neuroimaging experiments using predictions derived from these models involve learning about appetitive outcomes (juice, monetary rewards). However, fear conditioning is also prediction error driven (Laviolette and Grace, 2006; McNally and Westbrook, 2006; Cole and McNally, 2007, 2009; McNally et al., 2011), involving a circuit incorporating the ventral tegmental area (VTA), amygdala and hippocampus as well as the striatum and prefrontal cortex (Schiller et al., 2008) – all implicated in aberrant prediction error (see Corlett et al., 2007a for more detail). Descending opioids and NMDA receptors are also critically involved in predictive fear learning (McNally and Westbrook, 2006; Cole and McNally, 2007, 2009; McNally et al., 2011), neurotransmitters that may be compromised in schizophrenia (see below). NMDA receptor antagonism (itself a pharmacological model of psychosis; Krystal et al., 1994) modulates the degree to which prediction error signals contribute to belief updating, possibly through aberrant predictive learning (Corlett et al., 2010a). In contrast, the opioid antagonist naloxone modulates the actual prediction error signals during fear conditioning (McNally and Westbrook, 2006; Cole and McNally, 2007, 2009; McNally et al., 2011), perhaps through an interaction with VTA dopamine cells. Aberrations of these neurotransmitter processes could induce inappropriate fear following perceived cues, resulting in a subsequent elevation of uncertainty, both of which may contribute to delusion formation (Corlett et al., 2010a). Furthermore, individuals with a low tolerance for ambiguity are more prone to paranormal beliefs and odd experiences (Houran and Houran, 1998). One possibility is that intense emotional states and learning dysfunction contribute to a vicious circle in which fear and aberrant perception are mutually reinforcing and demand explanation (Pally, 2005, 2007), ultimately contributing to aberrant beliefs (Corlett et al., 2010a). Moreover, such processes could contribute to aberrant belief maintenance (see Box 2).

Box 2 **Delusions, emotions, and memory.**

It is adaptive for an organism to be able to remember salient events for extended periods without the necessity for repeat experiences (Dickinson, 2001). Reactivation of a memory trace for a salient event may increase the stabilization of that trace (Lee, 2008). Based on this hypothesis, our most salient memories would be reactivated most frequently and would therefore undergo greatest reconsolidation-based stabilization, increasing their fixity. Reconsolidation may facilitate the automation of behavior (Stickgold and Walker, 2007) – the transition from knowledge to belief (Eichenbaum and Bodkin, 2000) – shifting the representation that mediates behavior from declarative to procedural and thus reducing the demand for executive control. Further, reconsolidation is held to aid the extraction of important details from complex episodic memories and to permit the integration of those details in support of adaptive and efficient behaviors, possibly through the construction of habits or schemas (Bartlett, 1932). Our hypothesis is that delusions form under the influence of aberrant prediction errors, whose salience is anxiogenic and may demand explanation. Once delusions form, there may be relief from forming an explanation (Chouinard and Miller, 1999). In this proposal, subsequent aberrant salient experiences could reactivate the “explanation” and are interpreted in light of it and therefore strengthened via memory reconsolidation (Lee, 2008). Future work will be needed to further verify the role of stress, motivation and other emotional factors in this putative process.

We discussed the evidence regarding aberrant belief formation, but what about its maintenance? The state of a memory after consolidation may depend on the context in which it is recalled. Specifically, surprising information can update the memory trace or engage extinction learning, which competes with and overrides original traces (Pedreira et al., 2004). There is clinical phenomenological evidence consistent with the presence of competing representations, such as the duality of belief and disbelief during treatment (Stanton and David, 2000). Furthermore, delusions are elastic in the face of contradictory evidence (i.e., patients will incorporate contradictory evidence quite readily, strengthening the belief; Milton et al., 1978). The prognosis and response to cognitive behavioral therapy are worse for individuals engaging in this elasticity (Garety, 1991). There is further clinical evidence from a study involving the erasure of delusions following their engagement, administering electro-convulsive therapy following delusion reactivation (Rubin, 1976). More recently, there is cognitive behavioral evidence showing enhanced illusory truth effect in psychotic patients – when merely exposed to delusion congruent information, individuals with delusions will subsequently be more convinced of its truth when re-exposed to that information (Moritz et al., 2012). To summarize, patients may formulate an explanation for their aberrantly salient experiences. Once such a delusional scheme is formed it may be deployed in future contexts to explain subsequent aberrant experiences, strengthening the delusional association in the process. We recently discussed how the increasingly influential Bayesian brain account might help us to understand the role of prediction error in perception, belief, and delusion formation and maintenance (Corlett et al., 2009a; Fletcher and Frith, 2009). It will be key for future studies and theories to consider stress and emotional/motivational factors in the ongoing process of reconsolidation that may operate in delusion maintenance.

In considering how affect may contribute to delusion formation and maintenance through interaction with learning mechanisms, we highlight how overlapping and interactive neural processes may engender aberrant beliefs, similarly to other processes reviewed above. For instance, a failure of top-down regulation of the amygdala by prefrontal cortex may contribute to excessive and inappropriate fear responses (Corlett et al., 2011). One specific prediction of this interactive emotion-cognition model is that anxious individuals may become more paranoid in context of pharmacological manipulations that may exacerbate aberrant beliefs (i.e., NMDA receptor antagonists), which would derange the specification and incorporation of prediction error signals in a system that was already sensitized. Future work should explore this possibility. Furthermore, by taking a more reductionist approach grounded in formal animal learning theory, we attempt to provide a translational framework, linking psychological/cognitive accounts with basic neuroscience findings (e.g., fear-learning mechanisms in animals). We hope that future studies continue to delineate the interactive role of affect with learning and motivational mechanisms that may operate in the formation of aberrant beliefs.

Next, we discuss how emotion-cognition dysinteraction across described processes in schizophrenia might arise from hypothesized downstream disruptions in cellular-level computations and in turn impact large-scale inter-correlated brain systems that have been linked to symptoms.

## Understanding Affective and Cognitive Deficits in Schizophrenia Across Levels of Analyses

Thus far we have discussed the complexity of cognitive and emotional processes affected in schizophrenia. We have focused on evidence and theoretical understanding at the level of neural systems and behavior – levels of analyses typically examined using cognitive neuroscience approaches.

As reviewed, cognitive neuroscience has established the tools to probe the underlying circuitry that may be affected in schizophrenia, as well as its relationship to cognition and emotion. By studying pathological states using these tools, we gain insights about the neurobiological and psychological mechanisms through which impairments in cognition and emotion might contribute to psychiatric illness. However, such approaches have more difficulty identifying underlying cellular mechanisms (in humans, and therefore patients suffering from mental illness, at least). Such a step is crucial to identify effective pharmacological therapies. We believe it will be critical to close the existing gaps in our understanding of emotion and cognition in schizophrenia across levels of explanation: from synaptic signaling at the micro-circuit level, to system-level disruptions, and ultimately behavior. We acknowledge that a comprehensive review of the neurobiology and neurochemical alterations in schizophrenia is beyond the scope of this manuscript. However, we briefly highlight how evolving cellular-level hypotheses of mirco-circuit disruptions offer a possible foundation for understanding higher-order emergent neural system and behavioral deficits in schizophrenia that demand mechanistic explanations.

**Figure 2:**
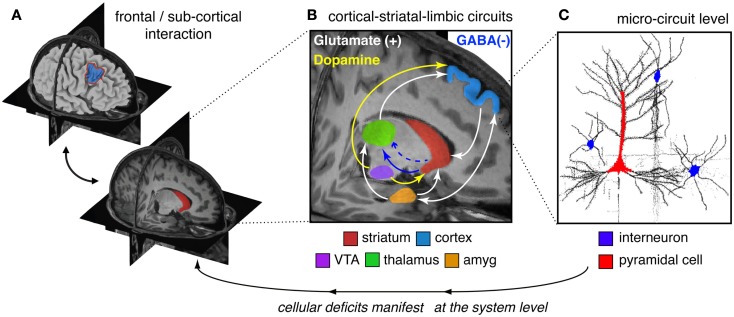
**Conceptual illustration of neural circuitry across levels of computation that may be involved in affective and cognitive disturbances in schizophrenia**. The figure highlights how, in order to explain deficits at the phenomenological/behavioral level, we need to bridge observations across multiple levels of analysis in schizophrenia. **(A)** At the regional level there is clear evidence for both structural and functional abnormalities in cortical (Csernansky et al., 1998, 2004, 2008; Harms et al., 2010, 2012) and striatal/thalamic circuits in schizophrenia (Csernansky et al., 1998, 2004, 2008; Laruelle et al., 1999; Harms et al., 2007; Reichenberg and Harvey, 2007; Dowd and Barch, 2010; Mamah et al., 2010). **(B)** However, less is known about how some of these regional deficits manifest in possible system-level disruptions in functional loops between prefrontal, striatal, limbic, and thalamic nodes in schizophrenia (Lisman, 2012). Deficits in these interacting functional systems need to be considered when interpreting abnormalities between affective/hot and cognitive/cold operations in schizophrenia. Furthermore, there is known interplay between interacting neurotransmitter systems in cortico-striatal-thalamic loops that may be compromised in schizophrenia (Carlsson et al., 2001; Coyle, 2006). **(C)** Based on emerging findings from basic animal (Lewis et al., 2004; Yizhar et al., 2011), post-mortem (Lewis et al., 2005, 2012), and pharmacological studies (Krystal et al., 2003), there is an increasing understanding of micro-circuit abnormalities that may be at play in schizophrenia (Marin, 2012). It may be possible that abnormalities in the balance of excitation/inhibition in cortical microcircuitry contribute to downstream system-level disturbances that encompass distributed circuits and neurotransmitter systems. One leading hypothesis postulates an imbalance between cortical excitation and inhibition between pyramidal cells (red) and interneurons (blue), producing a state of “disinhibition,” which may in turn affect regional and system-level function (Yizhar et al., 2011). Considering effects across all of these levels will be critical to mechanistically understand complex schizophrenia phenomenology and symptom-level interactions.

The clinical neuroscience approach to schizophrenia has identified region-level abnormalities in both function and anatomy across areas such as DLPFC, hippocampus, amygdala, thalamus, and striatum (Csernansky et al., 1998, 2004, 2008; Laruelle et al., 1999; Harms et al., 2007, 2010, 2012; Reichenberg and Harvey, 2007; Dowd and Barch, 2010; Mamah et al., 2010; Figure 2A). This is not to say that other regions are not affected – we simply use these as an illustrative example for how to link levels of understanding. However, regions such as DLPFC, striatum, amygdala, and thalamus comprise a set of cortical-subcortical networks and loops, which function in concert and are influenced by multiple neuromodulatory signaling pathways (Carlsson and Carlsson, 1990; Carlsson et al., 2001). It is likely that such network-level interaction in cortico-thalamo-striatal circuits are crucial for organizing computations that support complex cognitive processes such as working memory and motivation (Barch and Dowd, 2010), likely impacted by interactive neurotransmitter systems such as glutamate and dopamine (Laruelle et al., 2003; Figure 2B). Indeed, disruptions in numerous interacting neurotransmitter systems, including dopamine, GABA, and glutamate have been implicated in schizophrenia (Kegeles et al., 2000; Abi-Dargham et al., 2002; Krystal et al., 2003; Lewis et al., 2012). As noted, there is mounting evidence for dopaminergic alterations in the striatum of patients with schizophrenia which result in hyperactivity (Laruelle et al., 1999; Howes et al., 2012; for review see Laruelle et al., 2003). Patients also present with reduced prefrontal dopamine tone (Howes et al., 2012; in particular hypo-stimulation of D1 receptors in PFC; Abi-Dargham et al., 2002). Patients may also exhibit disruptions in glutamateric signaling at the NMDA receptor (Krystal et al., 2003), as well as disruptions in GABA synthesis and signaling from interneurons onto pyramidal cells (Lewis et al., 2004, 2005, 2012; Gonzalez-Burgos and Lewis, 2012; Nakazawa et al., 2012; Figure 2B). There is still an ongoing *chicken or the egg* debate as to which one of these disruptions may be the proximal cause of downstream symptoms (Coyle, 2006), yet considering these complex interactions will be vital as we move toward a more complete understanding of this illness.

One way to organize these multiple, interactive dysfunctions across levels of analysis is to consider how they may be impacted by pathology at the level of cortical microcircuitry (Figure 2C; Lisman, 2012; Lewis et al., 2012; Marin, 2012). That is, perhaps if we were to start from cellular-level hypotheses of disrupted cortical computations in schizophrenia, we may ultimately be able to better understand complex dynamics that emerge at higher levels of observation (Loh et al., 2007; Rolls and Deco, 2011; Lisman, 2012). Optimal cortical function depends on the balanced interaction of pyramidal excitatory (glutamatergic) and inhibitory (GABAergic) neurons (Shadlen and Newsome, 1994). Disruptions of this balance can have drastic behavioral consequences (Yizhar et al., 2011; Marin, 2012). In schizophrenia there may be a functional deficit in the interaction between excitatory and inhibitory cortical neurons (Benes et al., 1991; Lewis et al., 2004, 2005, 2012; Lewis and Moghaddam, 2006; Marin, 2012). This may arise from a disruption in cortical inhibition; stemming perhaps from reduced inhibitory drive via GABA interneurons onto pyramidal cells and ultimately resulting in *disinhibition* of pyramidal cells (Lewis et al., 2012; Marin, 2012). Post-mortem studies of patients with schizophrenia consistently show reduced levels of the mRNA for the 67-kD isoform of glutamic acid decarboxylase (GAD67, encoded by *GAD1*), a key factor in optimal GABA levels, in the DLPFC of patients with schizophrenia (for review see Lewis et al., 2005). Furthermore, GABA’s role in exerting lateral inhibition and synchronizing persistent firing of pyramidal cells in DLPFC (Rao et al., 2000) provides one potential mechanism for the tuning of representations of information. That is, lateral inhibition might enhance the processing and maintenance of salient information relative to less behaviorally relevant representations. Disruption of excitation/inhibitory (E/I) balance between pyramidal and GABA neurons may be one crucial pathophysiological mechanism operating in schizophrenia, relevant to the patterns of neural and behavioral responses that we discuss presently.

Therefore, perhaps a way of reconciling abnormalities in cognition/emotion and circuit-level disruptions is to consider a common computational motif: “attractor states.” Attractor states represent reverberating patterns of neural activity that are candidate mechanisms for working memory (outlined above Wang, 1999), perception (Braun and Mattia, 2010), and emotion (Rolls and Stringer, 2001). Attractor states and their sustaining recurrent neural activity are also crucial to some models of predictive coding in perception (Kiebel et al., 2009) and prediction error driven reinforcement learning in the basal ganglia (providing the reward expectation in a given situation; Morita et al., 2012). Neural evidence across species suggests that such attractor mechanisms exist. However, rather than remaining in a steady state, populations of neurons can jump from one attractor state to another (Hopfield, 1982), driven by intrinsic neural activity. Such intrinsic neural activity can actually contain meaningful spatial and temporal information and may encourage transitions across the attractor landscape (Braun and Mattia, 2010) – for example the prior expectations that constrain current perception (Berkes et al., 2011), which if mis-specified might result in psychotic symptoms (Corlett et al., 2009a). We argue that while cognitive, emotional, and delusional symptoms may appear “distinct,” they not only interact, but at a neural level, their pathophysiology may share important common features such that disruptions can affect all processes and the interaction between such processes. Thus, examples discussed above, where response patterns may be exaggerated following a “neutral” cue, could be considered in terms of the inappropriate establishment of an attractor state for that cue, possibly due to inappropriate function within a region (e.g., GABAergic disinhibition) or between regions (e.g., glutamate spillover and increased noise in message passing between regions; Yamashita and Tani, 2012).

We appreciate that, at present, it is unknown how these cellular disruptions in E/I balance may manifest at the level of neural systems and ultimately diverse psychological processes compromised in this illness (Yizhar et al., 2011). The challenge facing the field is to close this gap. There are paths forward: (i) one approach is to start at the level of cells and make predictions regarding the higher levels of analysis. A way to accomplish this goal could involve computational modeling (Montague et al., 2012), particularly models that are rooted in neurophysiologic data and that build on assumptions based on molecular and systems neuroscience (Wang, 2006, 2008, 2010; Anticevic et al., 2012a); (ii) another approach is to test hypotheses regarding neural dysfunction in schizophrenia via pharmacological manipulations in healthy adults (Corlett et al., 2007b). This is accomplished through perturbations of the underlying circuitry thought to be compromised in individuals suffering from the illness (Honey and Bullmore, 2004), via relatively well-understood neurochemical mechanisms. Furthermore, attempts have been made to unite the pharmacological and psychological levels of analysis to explain why these interventions are psychotomimetic (Corlett et al., 2007b; Anticevic et al., 2012a). In turn, such manipulations may reveal clues regarding specific links between disruptions in neurotransmitter systems, which can be connected to system-level deficits and ultimately behavior (Anticevic et al., 2012a); (iii) continued development of more sophisticated animal models of the neural pathology that may be present in this illness that are well-linked to both intermediate and behavioral phenotypic markers should also be pursued (Yizhar et al., 2011). For instance, primate physiology experiments of working memory, in combination with targeted neurochemical and optogenetic manipulations (Tye and Diesseroth, 2012), provide this framework (Simen et al., 2009; Arnsten, 2011; Arnsten and Rubia, 2012); (iv) and lastly, large-scale imaging genomic studies as well as detailed spatial and temporal genetic transcriptomics approaches (Johnson et al., 2009; Kang et al., 2011) could hone our search for genes that influence cortical development, that may ultimately disrupt the cortical microcircuitry detailed above. We argue that these and other complementary neuroscientific approaches will be critical to close our vast explanatory gaps in clinical neuroscience of schizophrenia and ultimately move toward ameliorating dysfunction in neural systems affecting cognition, emotion, and belief.

## Conclusion

To summarize, we discussed the interplay of cognition and emotion in schizophrenia across different processes – namely motivation and anhedonia, perceiving and filtering sensory stimuli, and how affective responses may interplay with learning mechanisms in the context of belief formation. We argue that, while some affective responses may be seemingly intact in schizophrenia, it is vital to consider how emotion and cognition impact upon one another across contexts to achieve a full understanding of emergent symptom-level disturbances in this illness. Finally, we discuss the evolving understanding of the underlying pathophysiology in schizophrenia. Here we argue that a translational perspective across levels of analyses – from cellular to system-level phenomena, and in turn behavioral deficits – will be critical to fully characterize the complex and debilitating symptoms observed in schizophrenia.

## Conflict of Interest Statement

The authors declare that the research was conducted in the absence of any commercial or financial relationships that could be construed as a potential conflict of interest.

## References

[B1] Abi-Dargham A. Mawlawi O. Lombardo I. Gil R. Martinez D. Huang Y. (2002). Prefrontal dopamine D1 receptors and working memory in schizophrenia. J. Neurosci. 22, 3708–3719 11978847 10.1523/JNEUROSCI.22-09-03708.2002 PMC6758376

[B2] Adams C. A. Dickinson A. (1981). Actions and habits: variations in associative representations during instrumental learning, in Information Processing in Animals: Memory Mechanisms, eds Spear N. E. Miller R. R. (Hillsdale: Lawrence Erlbaum).

[B3] Aleman A. Kahn R. S. (2005). Strange feelings: do amygdala abnormalities dysregulate the emotional brain in schizophrenia? Prog. Neurobiol. 77, 283–298 16352388 10.1016/j.pneurobio.2005.11.005

[B4] Anand A. Charney D. S. Oren D. A. Berman R. M. Hu X. S. Cappiello A. (2000). Attenuation of the neuropsychiatric effects of ketamine with lamotrigine: support for hyperglutamatergic effects of N-methyl-D-aspartate receptor antagonists. Arch. Gen. Psychiatry 57, 270–276 10.1001/archpsyc.57.3.270 10711913

[B5] Anticevic A. Gancsos M. Murray J. D. Repovs G. Driesen N. R. Ennis D. J. (2012a). NMDA receptor function in large-scale anti-correlated neural systems with implications for cognition and schizophrenia. Proc. Natl. Acad. Sci. U.S.A. 10.1073/pnas.1208494109 PMC3478611 23012427

[B6] Anticevic A. Repovs G. Barch D. M. (2012b). Emotion effects on attention, amygdala activation, and functional connectivity in schizophrenia. Schizophr. Bull. 38, 967–980 10.1093/schbul/sbq168 21415225 PMC3446234

[B7] Anticevic A. Van Snellenberg J. X. Cohen R. E. Repovs G. Dowd E. C. Barch D. M. (2012c). Amygdala recruitment in schizophrenia in response to aversive emotional material: a meta-analysis of neuroimaging studies. Schizophr. Bull. 38, 608–621 10.1093/schbul/sbq168 21123853 PMC3329999

[B8] Anticevic A. Repovs G. Barch D. M. (2011a). Working memory encoding and maintenance deficits in schizophrenia: neural evidence for activation and deactivation abnormalities. Schizophr. Bull. [Epub ahead of print]. 10.1093/schbul/sbr107 PMC3523909 21914644

[B9] Anticevic A. Repovs G. Corlett P. R. Barch D. M. (2011b). Negative and non-emotional interference with visual working memory in schizophrenia. Biol. Psychiatry 70, 1159–1168 10.1016/j.biopsych.2011.07.010 21861986

[B10] Arnsten A. F. (2011). Catecholamine influences on dorsolateral prefrontal cortical networks. Biol. Psychiatry 69, 89–99 10.1016/j.biopsych.2011.03.010 PMC3145207 21489408

[B11] Arnsten A. F. Rubia K. (2012). Neurobiological circuits regulating attention, cognitive control, motivation, and emotion: disruptions in neurodevelopmental psychiatric disorders. J. Am. Acad. Child Adolesc. Psychiatry 51, 356–367 10.1016/j.jaac.2012.01.008 22449642

[B12] Barbas H. (2007). Flow of information for emotions through temporal and orbitofrontal pathways. J. Anat. 237–249 10.1111/j.1469-7580.2007.00777.x 17635630 PMC2375774

[B13] Barch D. M. (2005). “The cognitive neuroscience of schizophrenia,” in Annual Review of Clinical Psychology, eds Cannon T. Mineka S. (Washington: American Psychological Association), 321–353 10.1146/annurev.clinpsy.1.102803.143959 17716091

[B14] Barch D. M. Carter C. S. Cohen J. D. (2003). Context processing deficit in schizophrenia: diagnostic specificity, 4-week course, and relationships to clinical symptoms. J. Abnorm. Psychol. 112, 132–143 10.1037/0021-843X.112.1.132 12653421

[B15] Barch D. M. Ceaser A. (2012). Cognition in schizophrenia: core psychological and neural mechanisms. Trends Cogn. Sci. 16, 27–34 10.1016/j.tics.2011.11.015 22169777 PMC3860986

[B16] Barch D. M. Dowd E. C. (2010). Goal representations and motivational drive in schizophrenia: the role of prefrontal-striatal interactions. Schizophr. Bull. 36, 919–934 10.1093/schbul/sbq068 20566491 PMC2930335

[B17] Barch D. M. Keefe R. S. E. (2010). Anticipating DSM-V: opportunities and challenges for cognition and psychosis. Schizophr. Bull. 36, 43–47 10.1093/schbul/sbq068 19923191 PMC2800153

[B18] Bartlett F. C. (1932). Remembering. Cambridge: Cambridge University Press

[B19] Benes F. M. McSparren J. Bird E. D. SanGiovanni J. P. Vincent S. L. (1991). Deficits in small interneurons in prefrontal and cingulate cortices of schizophrenic and schizoaffective patients. Arch. Gen. Psychiatry 48, 996–1001 10.1001/archpsyc.1991.01810350036005 1747023

[B20] Beninger R. J. (1983). The role of dopamine in locomotor activity and learning. Brain Res. 287, 173–196 6357357 10.1016/0165-0173(83)90038-3

[B21] Bentall R. P. Corcoran R. Howard R. Blackwood N. Kinderman P. (2001). Persecutory delusions: a review and theoretical integration. Clin. Psychol. Rev. 21, 1143–1192 10.1016/S0272-7358(01)00106-4 11702511

[B22] Bentall R. P. Swarbrick R. (2003). The best laid schemas of paranoid patients: autonomy, sociotropy and need for closure. Psychol. Psychother. 76(Pt 2), 163–171 10.1348/147608303765951195 12855062

[B23] Berenbaum H. Oltmanns T. F. (1992). Emotional experience and expression in schizophrenia and depression. J. Abnorm. Psychol. 101, 37–44 10.1037/0021-843X.101.1.37 1537971 PMC4370316

[B24] Berkes P. Orbán G. Lengyel M. Fiser J. (2011). Spontaneous cortical activity reveals hallmarks of an optimal internal model of the environment. Science 331, 83–87 10.1126/science.1195870 21212356 PMC3065813

[B25] Berridge K. C. (2003). Pleasures of the brain. Brain Cogn. 52, 106–128 10.1016/S0278-2626(03)00014-9 12812810

[B26] Berridge K. C. (2004). Motivation concepts in behavioral neuroscience. Physiol. Behav. 81, 179–209 10.1016/j.physbeh.2004.02.004 15159167

[B27] Bleuler E. (1911). Dementia Praecox, or The Group of Schizophrenias. New York: International Universities Press

[B28] Bradley M. M. Sabatinelli D. Lang P. J. Fitzsimmons J. R. King W. Desai P. (2003). Activation of the visual cortex in motivated attention. Behav. Neurosci. 117, 369–380 10.1037/0735-7044.117.2.369 12708533

[B29] Braun J. Mattia M. (2010). Attractors and noise: twin drivers of decisions and multistability. Neuroimage 52, 740–751 10.1016/j.neuroimage.2009.12.126 20083212

[B30] Braver T. S. (2012). The variable nature of cognitive control: a dual mechanisms framework. Trends Cogn. Sci. (Regul. Ed.) 16, 106–113 10.1016/j.tics.2011.12.010 22245618 PMC3289517

[B31] Braver T. S. Barch D. M. Cohen J. D. (1999). Cognition and control in schizophrenia: a computational model of dopamine and prefrontal function. Biol. Psychiatry 46, 312–328 10.1016/S0006-3223(99)00116-X 10435197

[B32] Burbridge J. A. Barch D. M. (2002). Emotional valence and reference disturbance in schizophrenia. J. Abnorm. Psychol. 111, 186–191 10.1037/0021-843X.111.1.186 11866172

[B33] Butts C. (1998). A Bayesian model of panic in belief. Comput. Math. Organ. Theory 4, 373–404 10.1023/A:1009638514137

[B34] Carlsson A. Waters N. Holm-Waters S. Tedroff J. Nilsson M. Carlsson M. L. (2001). Interactions between monoamines, glutamate, and GABA in schizophrenia: new evidence. Annu. Rev. Pharmacol. Toxicol. 41, 237–260 10.1146/annurev.pharmtox.41.1.237 11264457

[B35] Carlsson M. Carlsson A. (1990). Interactions between glutamatergic and monoaminergic systems within the basal ganglia – implications for schizophrenia and Parkinson’s disease. Trends Neurosci. 13, 272–276 10.1016/0166-2236(90)90108-M 1695402

[B37] Cedro A. Kokoszka A. Popiel A. Narkiewicz-Jodko W. (2001). Alexithymia in schizophrenia: an exploratory study. Psychol. Rep. 89, 95–98 10.2466/pr0.2001.89.1.95 11729558

[B38] Chen L. S. Rice T. K. Thompson P. A. Barch D. M. Csernansky J. G. (2009). Familial aggregation of clinical and neurocognitive features in sibling pairs with and without schizophrenia. Schizophr. Res. 111, 159–166 10.1016/j.schres.2009.03.030 19398304 PMC2813565

[B39] Chouinard G. Miller R. (1999). A rating scale for psychotic symptoms (RSPS) part I: theoretical principles and subscale 1: perception symptoms (illusions and hallucinations). Schizophr. Res. 38, 101–122 10.1016/S0920-9964(99)00012-2 10463458

[B40] Cole S. McNally G. P. (2007). Temporal-difference prediction errors and Pavlovian fear conditioning: role of NMDA and opioid receptors. Behav. Neurosci. 121, 1043–1052 10.1037/0735-7044.121.5.1043 17907835

[B41] Cole S. McNally G. P. (2009). Complementary roles for amygdala and periaqueductal gray in temporal-difference fear learning. Learn. Mem. 16, 1–7 10.1101/lm.1120509 19117910

[B42] Coltheart M. (2010). The neuropsychology of delusions. Ann. N. Y. Acad. Sci. 1191, 16–26 10.1111/j.1749-6632.2010.05496.x 20392273

[B43] Coltheart M. Langdon R. McKay R. (2011). Delusional belief. Annu. Rev. Psychol. 62, 271–298 10.1146/annurev.psych.121208.131622 20731601

[B44] Conrad K. (1958). Die BeginnendeSchizophrenie. Stuttgart: G. Thieme

[B45] Corlett P. R. Frith C. D. Fletcher P. C. (2009a). From drugs to deprivation: a Bayesian framework for understanding models of psychosis. Psychopharmacology (Berl.) 206, 515–530 10.1007/s00213-009-1561-0 19475401 PMC2755113

[B46] Corlett P. R. Krystal J. H. Taylor J. R. Fletcher P. C. (2009b). Why do delusions persist? Front. Hum. Neurosci. 3:12 10.3389/neuro.09.012.2009 19636384 PMC2713737

[B47] Corlett P. R. Honey G. D. Aitken M. R. Dickinson A. Shanks D. R. Absalom A. R. (2006). Frontal responses during learning predict vulnerability to the psychotogenic effects of ketamine: linking cognition, brain activity, and psychosis. Arch. Gen. Psychiatry 63, 611–621 10.1001/archpsyc.63.6.611 16754834

[B48] Corlett P. R. Honey G. D. Krystal J. H. Fletcher P. C. (2011). Glutamatergic model psychoses: prediction error, learning, and inference. Neuropsychopharmacology 36, 294–315 10.1038/npp.2010.163 20861831 PMC3055519

[B49] Corlett P. R. Murray G. K. Honey G. D. Aitken M. R. Shanks D. R. Robbins T. W. (2007a). Disrupted prediction-error signal in psychosis: evidence for an associative account of delusions. Brain 130(Pt 9), 2387–2400 10.1093/brain/awm173 17690132 PMC3838942

[B50] Corlett P. R. Honey G. D. Fletcher P. C. (2007b). From prediction error to psychosis: ketamine as a pharmacological model of delusions. J. Psychopharmacol. (Oxford) 21, 238–252 10.1177/0269881107077716 17591652

[B51] Corlett P. R. Taylor J. R. Wang X. J. Fletcher P. C. Krystal J. H. (2010a). Toward a neurobiology of delusions. Prog. Neurobiol. 92, 345–369 10.1016/j.pneurobio.2010.06.007 20558235 PMC3676875

[B52] Corlett P. R. D’Souza D. C. Krystal J. H. (2010b). Capgras syndrome induced by ketamine in a healthy subject. Biol. Psychiatry 68, e1–e2 10.1016/S0006-3223(10)00860-7 20385373 PMC3721067

[B53] Cornblatt B. Obuchowski M. Roberts S. Pollack S. Erlenmeyer-Kimling L. (1999). Cognitive and behavioral precursors of schizophrenia. Dev. Psychopathol. 11, 487–508 10.1017/S0954579499002175 10532621

[B54] Coyle J. T. (2006). Glutamate and schizophrenia: beyond the dopamine hypothesis. Cell. Mol. Neurobiol. 26, 365–384 10.1007/s10571-006-9062-8 16773445 PMC11881825

[B55] Csernansky J. G. Gillespie S. K. Dierker D. L. Anticevic A. Wang L. Barch D. M. (2008). Symmetric abnormalities in sulcal patterning in schizophrenia. Neuroimage 43, 440–446 10.1016/j.neuroimage.2008.07.034 18707008 PMC3011823

[B56] Csernansky J. G. Joshi S. Wang L. Haller J. W. Gado M. Miller J. P. (1998). Hippocampal morphometry in schizophrenia by high dimensional brain mapping. Proc. Natl. Acad. Sci. U.S.A. 95, 11406–11411 10.1073/pnas.95.19.11406 9736749 PMC21655

[B57] Csernansky J. G. Schindler M. K. Splinter N. R. Wang L. Gado M. Selemon L. D. (2004). Abnormalities of thalamic volume and shape in schizophrenia. Am. J. Psychiatry 161, 896–902 10.1176/appi.ajp.161.5.896 15121656

[B58] Daw N. D. Niv Y. Dayan P. (2005). Uncertainty-based competition between prefrontal and dorsolateral striatal systems for behavioral control. Nat. Neurosci. 8, 1704–1711 10.1038/nn1560 16286932

[B59] Delawalla Z. Barch D. M. Fisher Eastep J. L. Thomason E. S. Hanewinkel M. J. Thompson P. A. (2006). Factors mediating cognitive deficits and psychopathology among siblings of individuals with schizophrenia. Schizophr. Bull. 32, 525–537 10.1093/schbul/sbj082 16714471 PMC2632255

[B60] Dickinson A. (2001). The 28th Bartlett memorial lecture. Causal learning: an associative analysis. Q. J. Exp. Psychol. B 54, 3–25 10.1080/02724990042000010 11216300

[B61] Dickinson A. Balleine B. (1990). Motivational control of instrumental performance following a shift from thirst to hunger. Q. J. Exp. Psychol. B. 42, 413–431 2284440

[B62] Dickinson A. Smith J. Mirenowicz J. (2000). Dissociation of Pavlovian and instrumental incentive learning under dopamine antagonists. Behav. Neurosci. 114, 468–483 10.1037/0735-7044.114.3.468 10883798

[B63] Dolcos F. LaBar K. S. Cabeza R. (2004). Interaction between the amygdala and the medial temporal lobe memory system predicts better memory for emotional events. Neuron 42, 855–863 10.1016/S0896-6273(04)00289-2 15182723

[B64] Dowd E. C. Barch D. M. (2010). Anhedonia and emotional experience in schizophrenia: neural and behavioral indicators. Biol. Psychiatry 67, 902–911 10.1016/j.biopsych.2009.10.020 20004364 PMC3113677

[B65] Dutta R. Greene T. Addington J. McKenzie K. Phillips M. Murray R. M. (2007). Biological, life course, and cross-cultural studies all point toward the value of dimensional and developmental ratings in the classification of psychosis. Schizophr. Bull. 33, 868–876 10.1093/schbul/sbm059 17562692 PMC2632313

[B66] Edwards J. Jackson H. J. Pattison P. E. (2002). Emotion recognition via facial expression and affective prosody in schizophrenia: a methodological review. Clin. Psychol. Rev. 22, 789–832 10.1016/S0272-7358(02)00162-9 12214327

[B67] Eichenbaum H. Bodkin J. A. (2000). “Belief and knowledge as distinct forms of memory,” in Memory Brain and Belief, eds Schacter D. L. Scarry E. (Cambridge, MA: Harvard University Press), 176–207

[B68] Eisenhardt D. Menzel R. (2007). Extinction learning, reconsolidation and the internal reinforcement hypothesis. Neurobiol. Learn. Mem. 87, 167–173 10.1016/j.nlm.2006.09.005 17079171

[B69] Ellis H. D. Young A. W. (1990). Accounting for delusional misidentifications. Br. J. Psychiatry 157, 239–248 10.1192/bjp.157.2.239 2224375

[B70] Fletcher P. C. Frith C. D. (2009). Perceiving is believing: a Bayesian approach to explaining the positive symptoms of schizophrenia. Nat. Rev. Neurosci. 10, 48–58 10.1038/nrn2536 19050712

[B71] Fotopoulou A. (2010). The affective neuropsychology of confabulation and delusion. Cogn. Neuropsychiatry 15, 38–63 10.1080/13546800903250949 19823958

[B72] Gard D. E. Kring A. M. Gard M. G. Horan W. P. Green M. F. (2007). Anhedonia in schizophrenia: distinctions between anticipatory and consummatory pleasure. Schizophr. Res. 93, 253–260 10.1016/j.schres.2007.03.008 17490858 PMC1986826

[B73] Garety P. (1991). Reasoning and delusions. Br. J. Psychiatry Suppl. 14, 14–18 1840774

[B74] Glahn D. C. Ragland J. D. Abramoff A. Barrett J. Laird A. R. Bearden C. E. (2005). Beyond hypofrontality: a quantitative meta-analysis of functional neuroimaging studies of working memory in schizophrenia. Hum. Brain Mapp. 25, 60–69 10.1002/hbm.20138 15846819 PMC6871703

[B75] Goldman-Rakic P. S. Castner S. A. Svensson T. H. Siever L. J. Williams G. V. (2004). Targeting the dopamine D1 receptor in schizophrenia: insights for cognitive dysfunction. Psychopharmacology (Berl.) 174, 3–16 10.1007/s00213-004-1793-y 15118803

[B76] Gonzalez-Burgos G. Lewis D. A. (2012). NMDA receptor hypofunction, parvalbumin-positive neurons and cortical gamma oscillations in schizophrenia. Schizophr. Bull. [Epub ahead of print]. 10.1093/schbul/sbs010 22355184 PMC3446219

[B77] Gray J. A. (1995). Dopamine release in the nucleus accumbens: the perspective from aberrations of consciousness in schizophrenia. Neuropsychologia 33, 1143–1153 10.1016/0028-3932(95)00054-7 7501135

[B78] Gray J. A. (1998). Integrating schizophrenia. Schizophr. Bull. 24, 249–266 10.1093/oxfordjournals.schbul.a033324 9613624

[B79] Gray J. A. Feldon J. Rawlins J. N. P. Hemsley D. Smith A. D. (1991). The neuropsychology of schizophrenia. Behav. Brain Sci. 14, 1–84 10.1017/S0140525X0007237X

[B80] Gray J. A. Joseph M. H. Hemsley D. R. Young A. M. Warburton E. C. Boulenguez P. (1995). The role of mesolimbic dopaminergic and retrohippocampal afferents to the nucleus accumbens in latent inhibition: implications for schizophrenia. Behav. Brain Res. 71, 19–31 10.1016/0166-4328(95)00154-9 8747172

[B81] Green M. F. (2006). Cognitive impairment and functional outcome in schizophrenia and bipolar disorder. J. Clin. Psychiatry 67, e12 10.4088/JCP.0906e08 17107235

[B82] Greenwald A. G. Farnham S. D. (2000). Using the implicit association test to measure self-esteem and self-concept. J. Pers. Soc. Psychol. 79, 1022–1038 10.1037/0022-3514.79.6.1022 11138752

[B83] Greenwald A. G. McGhee D. E. Schwartz J. L. (1998). Measuring individual differences in implicit cognition: the implicit association test. J. Pers. Soc. Psychol. 74, 1464–1480 10.1037/0022-3514.74.6.1464 9654756

[B84] Gruzelier J. H. (1976). Clinical attributes of schizophrenic skin conductance responders and non-responders. Psychol. Med. 6, 254–249 10.1017/S0033291700013787 826921

[B85] Habel U. Gur R. C. Mandal M. K. Salloum J. B. Gur R. E. Schneider F. (2000). Emotional processing in schizophrenia across cultures: standardized measures of discrimination and experience. Schizophr. Res. 42, 57–66 10.1016/S0920-9964(99)00093-6 10706986

[B86] Hagen E. (2008). “Non-bizarre delusions as strategic deception,” in Medicine and Evolution: Current Applications, Future Prospect, eds Elton S. O’Higgins P. (New York: Taylor & Francis).

[B87] Harms M. P. Wang L. Campanella C. Aldridge K. Moffitt A. J. Kuelper J. (2010). Structural abnormalities in gyri of the prefrontal cortex in individuals with schizophrenia and their unaffected siblings. Br. J. Psychiatry 196, 150–157 10.1192/bjp.bp.109.067314 20118463 PMC2815937

[B88] Harms M. P. Wang L. Csernansky J. G. Barch D. M. (2012). Structure-function relationship of working memory activity with hippocampal and prefrontal cortex volumes. Brain Struct. Funct. [Epub ahead of print]. 10.1007/s00429-012-0391-8 22362200 PMC3858000

[B89] Harms M. P. Wang L. Mamah D. Barch D. M. Thompson P. A. Csernansky J. G. (2007). Thalamic shape abnormalities in individuals with schizophrenia and their nonpsychotic siblings. J. Neurosci. 27, 13835–13842 10.1523/JNEUROSCI.2571-07.2007 18077695 PMC6673612

[B90] Harvey P. O. Zaki J. Lee J. Ochsner K. Green M. F. (2012). Neural substrates of empathic accuracy in people with schizophrenia. Schizophr. Bull. [Epub ahead of print]. 10.1093/schbul/sbs042 PMC3627780 22451493

[B91] Heaton R. Gladsjo J. A. Palmer B. W. Kuck J. Marcotte T. D. Jeste D. V. (2001). Stability and course of neuropsychological deficits in schizophrenia. Arch. Gen. Psychiatry 58, 24–32 10.1001/archpsyc.58.1.24 11146755

[B92] Heckers S. Konradi C. (2010). Hippocampal pathology in schizophrenia. Curr. Top. Behav. Neurosci. 4, 529–553 10.1007/7854_2010_43 21312412

[B93] Heerey E. A. Gold J. M. (2007). Patients with schizophrenia demonstrate dissociation between affective experience and motivated behavior. J. Abnorm. Psychol. 116, 268–278 10.1037/0021-843X.116.2.268 17516760

[B94] Herbener E. S. Song W. Khine T. T. Sweeney J. A. (2008). What aspects of emotional functioning are impaired in schizophrenia? Schizophr. Res. 98, 239–246 10.1016/j.schres.2007.06.025 17689054 PMC2709502

[B95] Hitchcott P. K. Quinn J. J. Taylor J. R. (2007). Bidirectional modulation of goal-directed actions by prefrontal cortical dopamine. Cereb. Cortex 17, 2820–2827 10.1093/cercor/bhm010 17322558

[B96] Holt D. J. Lebron-Milad K. Milad M. R. Rauch S. L. Pitman R. K. Orr S. P. (2009). Extinction memory is impaired in schizophrenia. Biol. Psychiatry 65, 455–463 10.1016/j.biopsych.2008.09.017 18986648 PMC3740529

[B97] Honey G. Bullmore E. (2004). Human pharmacological MRI. Trends Pharmacol. Sci. 25, 366–374 10.1016/j.tips.2004.05.009 15219979

[B98] Hopfield J. J. (1982). Neural networks and physical systems with emergent collective computational abilities. Proc. Natl. Acad. Sci. U.S.A. 79, 2554–2558 10.1073/pnas.79.8.2554 6953413 PMC346238

[B99] Houran J. Houran J. (1998). Preliminary study of tolerance of ambiguity of individuals reporting paranormal experiences. Psychol. Rep. 82, 183 10.2466/pr0.1998.82.1.183 9520551

[B100] Howes O. D. Kambeitz J. Kim E. Stahl D. Slifstein M. Abi-Dargham A. (2012). The nature of dopamine dysfunction in schizophrenia and what this means for treatment: meta-analysis of imaging studies. Arch. Gen. Psychiatry 69, 776–786 10.1001/archgenpsychiatry.2012.169 22474070 PMC3730746

[B101] Howes O. D. Kapur S. (2009). The dopamine hypothesis of schizophrenia: version III – the final common pathway. Schizophr. Bull. 35, 549–562 10.1093/schbul/sbp006 19325164 PMC2669582

[B102] Howes O. D. Montgomery A. J. Asselin M. C. Murray R. M. Valli I. Tabraham P. (2009). Elevated striatal dopamine function linked to prodromal signs of schizophrenia. Arch. Gen. Psychiatry 66, 13–20 10.1001/archgenpsychiatry.2008.514 19124684

[B103] Insel T. R. (2010). Rethinking schizophrenia. Nature 468, 187–193 10.1038/nature09552 21068826

[B104] Insel T. R. Cuthbert B. N. (2009). Endophenotypes: bridging genomic complexity and disorder heterogeneity. Biol. Psychiatry 66, 988–989 10.1016/j.biopsych.2009.10.008 19900610

[B105] Irani F. Kalkstein S. Moberg E. A. Moberg P. J. (2011). Neuropsychological performance in older patients with schizophrenia: a meta-analysis of cross-sectional and longitudinal studies. Schizophr. Bull. 37, 1318–1326 10.1093/schbul/sbq057 20547571 PMC3196956

[B106] Iwase M. Yamashita K. Takahashi K. Kajimoto O. Shimizu A. Nishikawa T. (1999). Diminished facial expression despite the existence of pleasant emotional experience in schizophrenia. Methods Find Exp. Clin. Pharmacol. 21, 189–194 10.1358/mf.1999.21.3.534828 10389121

[B107] Jaspers K. (1963). General Psychopathology. Manchester: Manchester University Press

[B108] Jensen J. Willeit M. Zipursky R. B. Savina I. Smith A. J. Menon M. (2008). The formation of abnormal associations in schizophrenia: neural and behavioral evidence. Neuropsychopharmacology 33, 473–479 10.1038/sj.npp.1301437 17473838

[B109] Johnson M. R. Kawasawa Y. I. Mason C. E. Krsnik Z. Coppola G. Bogdanovic D. (2009). Functional and evolutionary insights into human brain development through global transcriptome analysis. Neuron 62, 494–509 10.1016/j.neuron.2009.03.027 19477152 PMC2739738

[B110] Jonides J. Lewis R. L. Nee D. E. Lustig C. A. Berman M. G. Moore K. S. (2008). The mind and brain of short-term memory. Annu. Rev. Psychol. 59, 193–224 10.1146/annurev.psych.59.103006.093615 17854286 PMC3971378

[B111] Juckel G. Schlagenhauf F. Koslowski M. Wüstenberg T. Villringer A. Knutson B. (2006a). Dysfunction of ventral striatal reward prediction in schizophrenia. Neuroimage 29, 409–416 10.1016/j.neuroimage.2005.07.051 16139525

[B112] Juckel G. Schlagenhauf F. Koslowski M. Filonov D. Wüstenberg T. Villringer A. (2006b). Dysfunction of ventral striatal reward prediction in schizophrenic patients treated with typical, not atypical, neuroleptics. Psychopharmacology (Berl.) 187, 222–228 10.1007/s00213-006-0405-4 16721614

[B113] Kaney S. Bentall R. P. (1989). Persecutory delusions and attributional style. Br. J. Med. Psychol. 62(Pt 2), 191–198 10.1111/j.2044-8341.1989.tb02826.x 2751948

[B114] Kaney S. Bentall R. P. (1992). Persecutory delusions and the self-serving bias. Evidence from a contingency judgment task. J. Nerv. Ment. Dis. 180, 773–780 10.1097/00005053-199212000-00006 1469376

[B115] Kang H. J. Kawasawa Y. I. Cheng F. Zhu Y. Xu X. Li M. (2011). Spatio-temporal transcriptome of the human brain. Nature 478, 483–489 10.1038/nature10523 22031440 PMC3566780

[B116] Kapur S. (2003). Psychosis as a state of aberrant salience: a framework linking biology, phenomenology, and pharmacology in schizophrenia. Am. J. Psychiatry 160, 13–23 10.1176/appi.ajp.160.1.13 12505794

[B117] Kapur S. Mizrahi R. Li M. (2005). From dopamine to salience to psychosis–linking biology, pharmacology and phenomenology of psychosis. Schizophr. Res. 79, 59–68 10.1016/j.schres.2005.01.003 16005191

[B118] Kee K. S. Green M. F. Mintz J. Brekke J. S. (2003). Is emotion processing a predictor of functional outcome in schizophrenia? Schizophr. Bull. 29, 487–497 10.1093/oxfordjournals.schbul.a007021 14609242

[B119] Kegeles L. S. Abi-Dargham A. Frankle W. G. Gil R. Cooper T. B. Slifstein M. (2010). Increased synaptic dopamine function in associative regions of the striatum in schizophrenia. Arch. Gen. Psychiatry 67, 231–239 10.1001/archgenpsychiatry.2010.10 20194823

[B120] Kegeles L. S. Abi-Dargham A. Zea-Ponce Y. Rodenhiser-Hill J. Mann J. J. Van Heertum R. L. (2000). Modulation of amphetamine-induced striatal dopamine release by ketamine in humans: implications for schizophrenia. Biol. Psychiatry 48, 627–640 10.1016/S0006-3223(00)00976-8 11032974

[B121] Kiebel S. J. Daunizeau J. Friston K. J. (2009). Perception and hierarchical dynamics. Front. Neuroinformatics 3:20 10.3389/neuro.11.020.2009 PMC2718783 19649171

[B122] Kinderman P. (1994). Attentional bias, persecutory delusions and the self-concept. Br. J. Med. Psychol. 67(Pt 1), 53–66 10.1111/j.2044-8341.1994.tb01770.x 8204542

[B123] Kinderman P. Bentall R. P. (1996). Self-discrepancies and persecutory delusions: evidence for a model of paranoid ideation. J. Abnorm. Psychol. 105, 106–113 10.1037/0021-843X.105.1.106 8666699

[B124] Knutson B. Westdorp A. Kaiser E. Hommer D. (2000). FMRI visualization of brain activity during a monetary incentive delay task. Neuroimage 12, 20–27 10.1006/nimg.2000.0593 10875899

[B125] Kober H. Barrett L. F. Joseph J. Bliss-Moreau E. Lindquist K. Wager T. D. (2008). Functional grouping and cortical-subcortical interactions in emotion: a meta-analysis of neuroimaging studies. Neuroimage 42, 998–1031 10.1016/j.neuroimage.2008.03.059 18579414 PMC2752702

[B126] Kohler C. G. Turner T. H. Bilker W. B. Brensinger C. M. Siegel S. J. Kanes S. J. (2003). Facial emotion recognition in schizophrenia: intensity effects and error pattern. Am. J. Psychiatry 160, 1768–1774 10.1176/appi.ajp.160.10.1768 14514489

[B127] Kraeplin E. (1950). Dementia Praecox and Paraphrenia. New York: International Universities Press, Inc

[B128] Krause R. Steimer E. Sänger-Alt C. Wagner G. (1989). Facial expression of schizophrenic patients and their interaction partners. Psychiatry 52, 1–12 2928411 10.1080/00332747.1989.11024424

[B129] Kring A. M. (2011). The future of emotion research in the study of psychopathology. Emot. Rev. 2, 225–228 10.1177/1754073910361986

[B130] Kring A. M. Alpert M. Neale J. M. Harvey P. D. (1994). A multimethod, multichannel assessment of affective flattening in schizophrenia. Psychiatry Res. 54, 211–222 10.1016/0165-1781(94)90008-6 7761554

[B131] Kring A. M. Kerr S. L. Smith D. A. Neale J. M. (1993). Flat affect in schizophrenia does not reflect diminished subjective experience of emotion. J. Abnorm. Psychol. 102, 507–517 10.1037/0021-843X.102.4.507 8282918

[B132] Kring A. M. Moran E. K. (2008). Emotional response deficits in schizophrenia: insights from affective science. Schizophr. Bull. 34, 819–834 10.1093/schbul/sbn071 18579556 PMC2632476

[B133] Kruglanski A. W. Webster D. M. (1996). Motivated closing of the mind: “seizing” and “freezing.” Psychol. Rev. 103, 263–283 10.1037/0033-295X.103.2.263 8637961

[B134] Kruglanski A. W. Webster D. M. Klem A. (1993). Motivated resistance and openness to persuasion in the presence or absence of prior information. J. Pers. Soc. Psychol. 65, 861–876 10.1037/0022-3514.65.5.861 8246114

[B135] Krystal J. H. D’Souza D. C. Karper L. P. Bennett A. Abi-Dargham A. Abi-Saab D. (1999). Interactive effects of subanesthetic ketamine and haloperidol in healthy humans. Psychopharmacology (Berl.) 145, 193–204 10.1007/s002130051049 10463321

[B136] Krystal J. H. D’Souza D. C. Mathalon D. Perry E. Belger A. Hoffman R. (2003). NMDA receptor antagonist effects, cortical glutamatergic function, and schizophrenia: toward a paradigm shift in medication development. Psychopharmacology (Berl.) 169, 215–233 10.1007/s00213-003-1582-z 12955285

[B137] Krystal J. H. Karper L. P. Seibyl J. P. Freeman G. K. Delaney R. Bremner J. D. (1994). Subanesthetic effects of the noncompetitive NMDA antagonist, ketamine, in humans. Psychotomimetic, perceptual, cognitive, and neuroendocrine responses. Arch. Gen. Psychiatry 51, 199–214 10.1001/archpsyc.1994.03950030035004 8122957

[B138] Krystal J. H. Perry E. B. Jr. Gueorguieva R. Belger A. Madonick S. H. Abi-Dargham A. (2005). Comparative and interactive human psychopharmacologic effects of ketamine and amphetamine: implications for glutamatergic and dopaminergic model psychoses and cognitive function. Arch. Gen. Psychiatry 62, 985–994 10.1001/archpsyc.62.9.985 16143730

[B139] Lang P. (2010). Emotion and motivation: toward consensus definitions and a common research purpose. Emot. Rev. 2, 229–233 10.1177/1754073910361984

[B140] Lang P. J. Bradley M. M. (2010). Emotion and the motivational brain. Biol. Psychol. 84, 437–450 10.1016/j.biopsycho.2009.10.007 19879918 PMC3612949

[B141] Lang P. J. Davis M. (2006). Emotion, motivation, and the brain: reflex foundations in animal and human research. Prog. Brain Res. 156, 3–29 10.1016/S0079-6123(06)56001-7 17015072

[B142] Laruelle M. Abi-Dargham A. Gil R. Kegeles L. Innis R. (1999). Increased dopamine transmission in schizophrenia: relationship to illness phases. Biol. Psychiatry 46, 56–72 10.1016/S0006-3223(99)00067-0 10394474

[B143] Laruelle M. Kegeles L. S. Abi-Dargham A. (2003). Glutamate, dopamine, and schizophrenia: from pathophysiology to treatment. Ann. N. Y. Acad. Sci. 1003, 138–158 10.1196/annals.1300.063 14684442

[B144] Laruelle M. VanDyck C. Abi-Dargham A. (1995). SPECT imaging of dopamine release following amphetamine challenge in healthy subjects and in patients with schizophrenia. J. Nucl. Med. 36, 10P 7790942

[B145] Laviolette S. R. Grace A. A. (2006). The roles of cannabinoid and dopamine receptor systems in neural emotional learning circuits: implications for schizophrenia and addiction. Cell. Mol. Life Sci. 63, 1597–1613 10.1007/s00018-006-6027-5 16699809 PMC11136137

[B146] LeDoux J. E. (2000). Emotion circuits in the brain. Annu. Rev. Neurosci. 23, 155–184 10.1146/annurev.neuro.23.1.155 10845062

[B147] Lee J. Park S. (2005). Working memory impairments in schizophrenia: a meta-analysis. J. Abnorm. Psychol. 114, 599–611 10.1037/0021-843X.114.4.599 16351383

[B148] Lee J. L. (2008). Memory reconsolidation mediates the strengthening of memories by additional learning. Nat. Neurosci. 11, 1264–1266 10.1038/nn2065 18849987

[B149] Lewis D. A. Curley A. A. Glausier J. R. Volk D. W. (2012). Cortical parvalbumin interneurons and cognitive dysfunction in schizophrenia. Trends Neurosci. 35, 57–67 10.1016/j.tins.2011.10.004 22154068 PMC3253230

[B150] Lewis D. A. Hashimoto T. (2007). Deciphering the disease process of schizophrenia: the contribution of cortical GABA neurons. Int. Rev. Neurobiol. 78, 109–131 10.1016/S0074-7742(06)78004-7 17349859

[B151] Lewis D. A. Hashimoto T. Volk D. W. (2005). Cortical inhibitory neurons and schizophrenia. Nat. Rev. Neurosci. 6, 312–324 10.1038/nrn1648 15803162

[B152] Lewis D. A. Moghaddam B. (2006). Cognitive dysfunction in schizophrenia: convergence of gamma-aminobutyric acid and glutamate alterations. Arch. Neurol. 63, 1372–1376 10.1001/archneur.63.2.288 17030651

[B153] Lewis D. A. Volk D. W. Hashimoto T. (2004). Selective alterations in prefrontal cortical GABA neurotransmission in schizophrenia: a novel target for the treatment of working memory dysfunction. Psychopharmacology (Berl.) 174, 143–150 10.1007/s00213-003-1673-x 15205885

[B154] Lisman J. (2012). Excitation, inhibition, local oscillations, or large-scale loops: what causes the symptoms of schizophrenia? Curr. Opin. Neurobiol. 22, 537–544 10.1016/j.conb.2011.10.018 22079494 PMC3302967

[B155] Loh M. Rolls E. T. Deco G. (2007). A dynamical systems hypothesis of schizophrenia. PLoS Comput. Biol. 3, e228 10.1371/journal.pcbi.0030228 17997599 PMC2065887

[B156] Mamah D. Conturo T. E. Harms M. P. Akbudak E. Wang L. McMichael A. R. (2010). Anterior thalamic radiation integrity in schizophrenia: a diffusion-tensor imaging study. Psychiatry Res. 183, 144–150 10.1016/j.pscychresns.2010.04.013 20619618 PMC3887223

[B157] Mandal M. K. Pandey R. Prasad A. B. (1998). Facial expressions of emotions and schizophrenia: a review. Schizophr. Bull. 24, 399–412 10.1093/oxfordjournals.schbul.a033335 9718632

[B158] Marin O. (2012). Interneuron dysfunction in psychiatric disorders. Nat. Rev. Neurosci. 13, 107–120 22251963 10.1038/nrn3155

[B159] Mathews J. R. Barch D. M. (2004). Episodic memory for emotional and nonemotional words in schizophrenia. Cogn. Emot. 18, 721–740 10.1080/02699930341000284

[B160] Mattes R. M. Schneider F. Heimann H. Birbaumer N. (1995). Reduced emotional response of schizophrenic patients in remission during social interaction. Schizophr. Res. 17, 249–255 10.1016/0920-9964(95)00014-3 8664204

[B161] McKay R. Langdon R. Coltheart M. (2007a). Jumping to delusions? Paranoia, probabilistic reasoning, and need for closure. Cogn. Neuropsychiatry 12, 362–376 10.1080/13546800500363996 17558643

[B162] McKay R. Langdon R. Coltheart M. (2007b). Models of misbelief: integrating motivational and deficit theories of delusions. Conscious. Cogn. 16, 932–941 10.1016/j.concog.2006.06.001 17331741

[B163] McKay R. Langdon R. Coltheart M. (2007c). The defensive function of persecutory delusions: an investigation using the implicit association test. Cogn. Neuropsychiatry 12, 1–24 10.1080/13546800500363996 17162444

[B164] McKay R. T. Dennett D. C. (2009). The evolution of misbelief. Behav. Brain Sci. 32, 493–510; discussion 510–561. 10.1017/S0140525X09991555 20105353

[B165] McNally G. P. Johansen J. P. Blair H. T. (2011). Placing prediction into the fear circuit. Trends Neurosci. 34, 283–292 10.1016/j.tins.2011.03.005 21549434 PMC4245078

[B166] McNally G. P. Westbrook R. F. (2006). Predicting danger: the nature, consequences, and neural mechanisms of predictive fear learning. Learn. Mem. 13, 245–253 10.1101/lm.196606 16741278 PMC10807866

[B167] Mijovic-Prelec D. Prelec D. (2010). Self-deception as self-signalling: a model and experimental evidence. Philos. Trans. R. Soc. Lond. B Biol. Sci. 365, 227–240 10.1098/rstb.2009.0218 20026461 PMC2827460

[B168] Miller B. T. D’Esposito M. (2005). Searching for “the top” in top-down control. Neuron 48, 535–538 10.1016/j.neuron.2005.11.006 16301170

[B169] Miller E. K. Cohen J. D. (2001). An integrative theory of prefrontal cortex function. Annu. Rev. Neurosci. 24, 167–202 10.1146/annurev.neuro.24.1.167 11283309

[B170] Milton F. Patwa V. K. Hafner R. J. (1978). Confrontation vs. belief modification in persistently deluded patients. Br. J. Med. Psychol. 51, 127–130 10.1111/j.2044-8341.1978.tb02456.x 646958

[B171] Mishara A. L. Corlett P. R. (2009). Are delusions biologically adaptive? Salvaging the doxastic shear pin. Behav. Brain Sci. 32, 530–531 10.1017/S0140525X09991464

[B172] Montague P. R. Dolan R. J. Friston K. J. Dayan P. (2012). Computational psychiatry. Trends Cogn. Sci. (Regul. Ed.) 16, 72–80 10.1016/j.tics.2012.04.003 22177032 PMC3556822

[B173] Morita K. Morishima M. Sakai K. Kawaguchi Y. (2012). Reinforcement learning: computing the temporal difference of values via distinct corticostriatal pathways. Trends Neurosci. 35, 457–467 10.1016/j.tins.2012.04.009 22658226

[B174] Moritz S. Kother U. Woodward T. S. Veckenstedt R. Dechene A. Stahl C. (2012). Repetition is good? An internet trial on the illusory truth effect in schizophrenia and nonclinical participants. J. Behav. Ther. Exp. Psychiatry 43, 1058–1063 10.1016/j.jbtep.2012.04.004 22683551

[B175] Murray G. K. Corlett P. R. Clark L. Pessiglione M. Blackwell A. D. Honey G. (2008). Substantia nigra/ventral tegmental reward prediction error disruption in psychosis. Mol. Psychiatry 13, 239, 267–276. 10.1038/sj.mp.4002157 17684497 PMC2564111

[B176] Nakazawa K. Zsiros V. Jiang Z. Nakao K. Kolata S. Zhang S. (2012). GABAergic interneuron origin of schizophrenia pathophysiology. Neuropharmacology 62, 1574–1583 10.1016/j.neuropharm.2011.01.022 21277876 PMC3090452

[B177] Niedenthal P. M. Brauer M. (2012). Social functionality of human emotion. Annu. Rev. Psychol. 63, 259–285 10.1146/annurev.psych.121208.131605 22017377

[B178] Nielsen M. O. Rostrup E. Wulff S. Bak N. Lublin H. Kapur S. (2012). Alterations of the brain reward system in antipsychotic naïve schizophrenia patients. Biol. Psychiatry 71, 898–905 10.1016/j.biopsych.2012.02.007 22418013

[B179] Niendam T. A. Bearden C. E. Rosso I. M. Sanchez L. E. Hadley T. Nuechterlein K. H. (2003). A prospective study of childhood neurocognitive functioning in schizophrenic patients and their siblings. Am. J. Psychiatry 160, 2060–2062 10.1176/appi.ajp.160.11.2060 14594759

[B180] Ochsner K. N. (2008). The social-emotional processing stream: five core constructs and their translational potential for schizophrenia and beyond. Biol. Psychiatry 64, 48–61 10.1016/j.biopsych.2008.04.024 18549876 PMC2453243

[B181] O’Doherty J. P. Deichmann R. Critchley H. D. Dolan R. J. (2002). Neural responses during anticipation of a primary taste reward. Neuron 33, 815–826 10.1016/S0896-6273(02)00603-7 11879657

[B182] O’Tuathaigh C. M. Salum C. Young A. M. Pickering A. D. Joseph M. H. Moran P. M. (2003). The effect of amphetamine on Kamin blocking and overshadowing. Behav. Pharmacol. 14, 315–322 10.1097/01.fbp.0000080416.18561.3e 12838037

[B183] Owen A. M. McMillan K. M. Laird A. R. Bullmore E. (2005). N-back working memory paradigm: a meta-analysis of normative functional neuroimaging studies. Hum. Brain Mapp. 25, 46–59 10.1002/hbm.20131 15846822 PMC6871745

[B184] Padmala S. Pessoa L. (2008). Affective learning enhances visual detection and responses in primary visual cortex. J. Neurosci. 28, 6202–6210 10.1523/JNEUROSCI.1233-08.2008 18550762 PMC2673575

[B185] Pally R. (2005). Non-conscious prediction and a role for consciousness in correcting prediction errors. Cortex 41, 643–662; discussion 731–734. 10.1016/S0010-9452(08)70282-X 16209328

[B186] Pally R. (2007). The predicting brain: unconscious repetition, conscious reflection and therapeutic change. Int. J. Psychoanal. 88(Pt 4), 861–881 10.1516/B328-8P54-2870-P703 17681897

[B187] Pearce J. M. Hall G. (1980). A model for Pavlovian learning: variations in the effectiveness of conditioned but not of unconditioned stimuli. Psychol. Rev. 87, 532–552 10.1037/0033-295X.87.6.532 7443916

[B188] Pedreira M. E. Perez-Cuesta L. M. Maldonado H. (2004). Mismatch between what is expected and what actually occurs triggers memory reconsolidation or extinction. Learn. Mem. 11, 579–585 10.1101/lm.76904 15466312 PMC523076

[B189] Peralta V. Cuesta M. J. (2001). How many and which are the psychopathological dimensions in schizophrenia? Issues influencing their ascertainment. Schizophr. Res. 49, 269–285 10.1016/S0920-9964(00)00071-2 11356588

[B190] Pessoa L. (2008). On the relationship between emotion and cognition. Nat. Rev. Neurosci. 9, 148–158 10.1038/nrn2317 18209732

[B191] Pessoa L. Adolphs R. (2010). Emotion processing and the amygdala: from a ‘low road’ to ‘many roads’ of evaluating biological significance. Nat. Rev. Neurosci. 11, 773–783 10.1038/nrn2920 20959860 PMC3025529

[B192] Petersen S. E. Snyder A. Z. Raichle M. E. (1990). Activation of extrastriate and frontal cortical areas by visual words and word-like stimuli. Science 249, 1041–1044 10.1126/science.2396097 2396097

[B193] Phillips M. L. (2003). Understanding the neurobiology of emotion perception: implications for psychiatry. Br. J. Psychiatry 182, 190–192 10.1192/bjp.182.3.190 12611778

[B194] Phillips M. L. Drevets W. C. Rauch S. L. Lane R. (2003). Neurobiology of emotion perception II: implications for major psychiatric disorders. Biol. Psychiatry 54, 515–528 10.1016/S0006-3223(03)00168-9 12946880

[B195] Posner M. I. Petersen S. E. (1990). The attention system of the human brain. Annu. Rev. Neurosci. 13, 25–42 10.1146/annurev.ne.13.030190.000325 2183676

[B196] Postle B. R. (2005). Delay-period activity in the prefrontal cortex: one function is sensory gating. J. Cogn. Neurosci. 17, 1679–1690 10.1162/089892905774589208 16269105 PMC1343532

[B197] Ramachandran V. Blakeslee S. (1998). Phantoms in the Brain: Probing the Mysteries of the Human Mind. New York: William Morrow

[B198] Rao S. G. Williams G. V. Goldman-Rakic P. S. (2000). Destruction and creation of spatial tuning by disinhibition: GABA(A) blockade of prefrontal cortical neurons engaged by working memory. J. Neurosci. 20, 485–494 10627624 10.1523/JNEUROSCI.20-01-00485.2000 PMC6774140

[B199] Reichenberg A. Harvey P. D. (2007). Neuropsychological impairments in schizophrenia: integration of performance-based and brain imaging findings. Psychol. Bull. 133, 833–858 10.1037/0033-2909.133.5.833 17723032

[B200] Rescorla R. A. Wagner A. R. (1972). “A theory of Pavlovian conditioning: variations in the effectiveness of reinforcement and non-reinforcement,” in Classical Conditioning II: Current Research and Theory, eds Black A. H. Prokasy W. F. (New York: Appleton-Century-Crofts), 64–99

[B201] Robinson T. E. Berridge K. C. (2001). Incentive-sensitization and addiction. Addiction 96, 103–114 10.1046/j.1360-0443.2001.9611038.x 11177523

[B202] Rolls E. T. Deco G. (2011). A computational neuroscience approach to schizophrenia and its onset. Neurosci. Biobehav. Rev. 35, 1644–1653 10.1016/j.neubiorev.2010.09.001 20851143

[B203] Rolls E. T. Stringer S. M. (2001). A model of the interaction between mood and memory. Network 12, 89–109 10.1080/713663216 11405424

[B204] Romaniuk L. Honey G. D. King J. R. Whalley H. C. McIntosh A. M. Levita L. (2010). Midbrain activation during pavlovian conditioning and delusional symptoms in schizophrenia. Arch. Gen. Psychiatry 67, 1246–1254 10.1001/archgenpsychiatry.2010.169 21135324

[B205] Ross C. A. Margolis R. L. Reading S. A. Pletnikov M. Coyle J. T. (2006). Neurobiology of schizophrenia. Neuron 52, 139–153 10.1016/j.neuron.2006.09.015 17015232

[B206] Rubin R. D. (1976). Clinical use of retrograde amnesia produced by electroconvulsive shock. A conditioning hypothesis. Can. Psychiatr. Assoc. J. 21, 87–90 1277097 10.1177/070674377602100205

[B207] Salzman C. D. Fusi S. (2010). Emotion, cognition, and mental state representation in amygdala and prefrontal cortex. Annu. Rev. Neurosci. 33, 173–202 10.1146/annurev.neuro.051508.135256 20331363 PMC3108339

[B208] Schiller D. Levy I. Niv Y. LeDoux J. E. Phelps E. A. (2008). From fear to safety and back: reversal of fear in the human brain. J. Neurosci. 28, 11517–11525 10.1523/JNEUROSCI.2265-08.2008 18987188 PMC3844784

[B209] Schlagenhauf F. Juckel G. Koslowski M. Kahnt T. Knutson B. Dembler T. (2008). Reward system activation in schizophrenic patients switched from typical neuroleptics to olanzapine. Psychopharmacology (Berl.) 196, 673–684 10.1007/s00213-007-1016-4 18097655

[B210] Scholten M. R. Aleman A. Montagne B. Kahn R. S. (2005). Schizophrenia and processing of facial emotions: sex matters. Schizophr. Res. 78, 61–67 10.1016/j.schres.2005.06.019 16084696

[B211] Shadlen M. N. Newsome W. T. (1994). Noise, neural codes and cortical organization. Curr. Opin. Neurobiol. 4, 569–579 10.1016/0959-4388(94)90059-0 7812147

[B212] Simen A. A. DiLeone R. Arnsten A. F. (2009). Primate models of schizophrenia: future possibilities. Prog. Brain Res. 179, 117–125 10.1016/S0079-6123(09)17913-X 20302824 PMC2929764

[B213] Sison C. E. Alpert M. Fudge R. Stern R. M. (1996). Constricted expressiveness and psychophysiological reactivity in schizophrenia. J. Nerv. Ment. Dis. 184, 589–597 10.1097/00005053-199610000-00002 8917155

[B214] Stanton B. David A. (2000). First-person accounts of delusions. Psychiatr. Bull. R. Coll. Psychiatr. 24, 333–336 10.1192/pb.24.9.333

[B215] Stephan K. E. Baldeweg T. Friston K. J. (2006). Synaptic plasticity and dysconnection in schizophrenia. Biol. Psychiatry 59, 929–939 10.1016/j.biopsych.2005.10.005 16427028

[B216] Stickgold R. Walker M. P. (2007). Sleep-dependent memory consolidation and reconsolidation. Sleep Med. 8, 331–343 10.1016/j.sleep.2007.03.011 17470412 PMC2680680

[B217] Sutton R. S. Barto A. G. (1998). Reinforcement Learning: An Introduction. Cambridge: MIT Press

[B218] Taylor S. Kang J. Brege I. S. Tso I. F. Hosanagar A. Johnson T. D. (2011). Meta-analysis of functional neuroimaging studies of emotion perception and experience in schizophrenia. Biol. Psychiatry 71, 136–145 10.1016/j.biopsych.2011.09.007 21993193 PMC3237865

[B219] Trémeau F. (2006). A review of emotion deficits in schizophrenia. Dialogues Clin. Neurosci. 8, 59–70 16640115 10.31887/DCNS.2006.8.1/ftremeau PMC3181757

[B220] Trémeau F. Malaspina D. Duval F. Corrêa H. Hager-Budny M. Coin-Bariou L. (2005). Facial expressiveness in patients with schizophrenia compared to depressed patients and nonpatient comparison subjects. Am. J. Psychiatry 162, 92–101 10.1176/appi.ajp.162.1.92 15625206

[B221] Tricomi E. Balleine B. W. O’Doherty J. P. (2009). A specific role for posterior dorsolateral striatum in human habit learning. Eur. J. Neurosci. 29, 2225–2232 10.1111/j.1460-9568.2009.06796.x 19490086 PMC2758609

[B222] Trivers R. (1985). Social Evolution. Menlo Park, CA: Benjamin/Cummins Publishing Co

[B223] Tulving E. (1972). “Episodic and semantic memory,” in Organization of Memory, eds Tulving E. Donaldson W. (New York: Academic Press), 381–403

[B224] Tye K. M. Diesseroth K. (2012). Optogenetic investigation of neural circuits underlying brain disease in animal models. Nat. Rev. Neurosci. 13, 251–266 10.1038/nrm3311 22430017 PMC6682316

[B225] Ursu S. Kring A. M. Gard M. G. Minzenberg M. J. Yoon J. H. Ragland J. D. (2011). Prefrontal cortical deficits and impaired cognition-emotion interactions in schizophrenia. Am. J. Psychiatry 168, 276–285 10.1176/appi.ajp.2010.09081215 21205806 PMC4019338

[B226] Van Snellenberg J. X. Torres I. J. Thornton A. E. (2006). Functional neuroimaging of working memory in schizophrenia: task performance as a moderating variable. Neuropsychology 20, 497–510 10.1037/0894-4105.20.5.497 16938013

[B227] Wager T. D. Smith E. E. (2003). Neuroimaging studies of working memory: a meta-analysis. Cogn. Affect. Behav. Neurosci. 3, 255–274 10.3758/CABN.3.4.255 15040547

[B228] Walker E. Kestler L. Bollini A. Hochman K. M. (2004). Schizophrenia: etiology and course. Annu. Rev. Psychol. 55, 401–430 10.1146/annurev.psych.55.090902.141950 14744221

[B229] Wang X. J. (1999). Synaptic basis of cortical persistent activity: the importance of NMDA receptors to working memory. J. Neurosci. 19, 9587–9603 10531461 10.1523/JNEUROSCI.19-21-09587.1999 PMC6782911

[B230] Wang X.-J. (2006). Toward a prefrontal microcircuit model for cognitive deficits in schizophrenia. Pharmacopsychiatry 39(Suppl. 1), S80–S87 10.1055/s-2006-931501 16508903

[B231] Wang X.-J. (2008). Decision making in recurrent neuronal circuits. Neuron 60, 215–234 10.1016/j.neuron.2008.09.034 18957215 PMC2710297

[B232] Wang X.-J. (2010). Neurophysiological and computational principles of cortical rhythms in cognition. Physiol. Rev. 90, 1195–1268 10.1152/physrev.00035.2008 20664082 PMC2923921

[B233] Whitson J. A. Galinsky A. D. (2008). Lacking control increases illusory pattern perception. Science 322, 115–117 10.1126/science.1159845 18832647

[B234] Yamashita Y. Tani J. (2012). Spontaneous prediction error generation in schizophrenia. PLoS ONE 7, e37843 10.1371/journal.pone.0037843 22666398 PMC3364276

[B235] Yizhar O. Fenno L. E. Prigge M. Schneider F. Davidson T. J. O’Shea D. J. (2011). Neocortical excitation/inhibition balance in information processing and social dysfunction. Nature 477, 171–178 10.1038/nature10360 21796121 PMC4155501

